# Divergent responses of native predators to severe wildfire and biological invasion are mediated by life history

**DOI:** 10.1002/eap.70135

**Published:** 2025-11-10

**Authors:** Joshua M. Barry, Connor M. Wood, Gavin M. Jones, Kate A. McGinn, Kevin G. Kelly, H. Anu Kramer, Daniel F. Hofstadter, Stefan Kahl, Holger Klinck, Nicholas F. Kryshak, Brian P. Dotters, Kevin N. Roberts, John J. Keane, Elizabeth Ng, M. Zachariah Peery

**Affiliations:** ^1^ Department of Forest and Wildlife Ecology University of Wisconsin‐Madison Madison Wisconsin USA; ^2^ K. Lisa Yang Center for Conservation Bioacoustics Cornell Lab of Ornithology, Cornell University Ithaca New York USA; ^3^ U.S. Forest Service – Rocky Mountain Research Station Albuquerque New Mexico USA; ^4^ Chemnitz University of Technology Chemnitz Germany; ^5^ Sierra Pacific Industries Anderson California USA; ^6^ US Department of Agriculture (USDA) Forest Service Pacific Southwest Research Station Placerville California USA

**Keywords:** barred owl, bioacoustics, disturbance, invasive species, Sierra Nevada, site occupancy, Strigidae, wildfire

## Abstract

The Anthropocene is defined by rapid environmental changes such as biological invasions and shifting disturbance regimes that threaten native species. Understanding the drivers of endangerment for species facing multiple simultaneous threats is challenging without experimental methods. Here, we examined the relative and combined effects of severe wildfires and an early‐stage barred owl (*Strix varia*) invasion on an assemblage of three native forest owl species in the Sierra Nevada, California, USA, leveraging manipulative (lethal barred owl removals) and natural (severe wildfires) experiments and a regional passive acoustic monitoring program from 2018 to 2023. Wildfires reduced flammulated owl (*Psiloscops flammeolus*) occupancy by 71% in severely burned areas (sites experiencing near‐complete high‐severity fire) for at least 3 years postfire but did not affect great horned (*Bubo virginianus*) or northern pygmy owl (*Glaucidium californicum*) occupancy. Because flammulated owls have small home ranges and an insectivorous diet that depends on nearby mature forest foraging habitat and secondary‐cavity nest sites, they showed a strong negative response to extensive high‐severity burn areas that eliminate these resources. Flammulated owl occupancy increased approximately twofold from 0.09 (85% CI: 0.03, 0.20) to 0.18 (85% CI: 0.07, 0.36) following lethal barred owl removals (with only 4% posterior distribution overlap), but removals did not affect the other two native species. Despite evidence of habitat segregation between barred owls and the native species, where barred owls typically occupied intermediate‐to‐late seral forests in flatter, lower elevation areas, this niche partitioning was insufficient to prevent nonconsumptive or predatory effects on flammulated owls. In contrast, the resilience of great horned and pygmy owls may have stemmed from their larger body size and diurnal activity, respectively, suggesting that life history mediates forest owl vulnerability to invasive barred owls. The negative effects of barred owls on flammulated owls, even during the early invasion stage, coupled with well‐documented effects on other small, nocturnal forest owl species in regions with high barred owl densities, reinforce the conservation value of proactive invasive species management. Our study demonstrates the power of regional‐scale experimentation, facilitated by bioacoustic monitoring, for understanding biological community responses—mediated by species' life history—to rapid environmental changes.

## INTRODUCTION

The Anthropocene is defined by rapid environmental changes that threaten biodiversity with uncertain outcomes (Turner & Seidl, 2023; Williams & Jackson, 2007). Among these changes, amplified disturbance regimes and biological invasions pose particularly acute threats as each can substantially alter the composition and function of biological communities (Simberloff et al., 2013; Stephens et al., 2014). Understanding the ecological effects of these stressors is challenging because disturbance regimes change over broad spatial scales and are inherently variable (Senf & Seidl, 2021), while the effects of invasive species depend on the invasion stage (Ricciardi, 2003; Strayer et al., 2006), landscape features promoting niche partitioning and coexistence (Blubaugh et al., 2022; Zwerschke et al., 2018), and the nature of species interactions (Alakoski et al., 2019). Moreover, many ecosystems are exposed to both shifting disturbance regimes and biological invasions, further challenging our understanding of the relative and combined ecological effects of these stressors (Paine et al., 1998; Turner et al., 2020). Indeed, co‐occurring stressors can yield a variety of effects on ecosystems, ranging from minimal to additive to synergistic (Harvey et al., 2013; Metz et al., 2013; Veblen et al., 1994), depending on the timing, type, and order of successive disturbances (Johnstone et al., 2016). Yet, understanding these potentially complex effects is essential for developing strategies for effective biodiversity conservation.

North American forests are experiencing rapidly changing wildfire regimes (Hagmann et al., 2021). Historically, many of these forests burned at subdecadal to decadal frequencies with low or mixed severities, creating heterogeneous landscapes characterized by a fine‐scale mosaic of forest ages and tree species (Hessburg et al., 2005; Scholl & Taylor, 2010). However, wildfires are growing in severity and size due to warming and drying climates, the legacy of forest management practices such as fire suppression and large tree harvesting, and the expansion of the wildland–urban interface, which has increased human‐caused ignitions (Balch et al., 2017; Knapp et al., 2013; Westerling, 2016). Concern over the effects of large, severe wildfires (i.e., “megafires”) has catalyzed many studies exploring how wildlife responds to wildfires (Fontaine & Kennedy, 2012). For example, diurnal avian communities, which have been extensively studied in dry western forests, display greater species diversity in heterogeneous forests with varied wildfire severities compared to large patches of severely burned or densely vegetated forests (Beedy, 1981; Burnett & Roberts, 2015; Steel et al., 2021; Tingley et al., 2016). In contrast, bat species diversity can be greater in areas burned by moderate‐to‐high severity wildfires compared to unburned forests, possibly due to the increased postfire availability of prey and shelter for many open‐forest adapted species (Blakey et al., 2019; Buchalski et al., 2013; Steel et al., 2019). Some pond‐breeding amphibians may withstand the immediate effects of severe wildfire by finding local refuges, while certain reptiles may opportunistically use burned areas for hunting or perching (Hossack & Corn, 2007). While the ecosystem services provided by nocturnal avian communities, particularly owls, have been recognized (Whelan et al., 2008), our understanding of their response to severe wildfires is primarily concentrated on a single species, the spotted owl (*Strix occidentalis*) (Jones et al., 2016; Kramer et al., 2021; Rockweit et al., 2017). Thus, additional information is necessary to support the conservation of biodiversity within this ecologically important taxon as resource agencies develop forest management strategies to reduce severe wildfire risks.

As megafires continue to increase in frequency in western North America, forest owls in this region are facing a new threat from the invasion of the barred owl (*Strix varia*), an apex predator from eastern North America that has recently expanded its range westward (Dunbar et al., 1991). The barred owl's range expansion is believed to be driven by postcolonial changes in land use, including increased forest cover in riparian zones, tree planting, the eradication of bison, and fire exclusion in the northern Great Plains (Livezey, 2009), coupled with warmer temperatures aiding in overcoming harsh winters. Invasive barred owls have established dense populations in much of the Pacific Northwest, where their large body size and flexible diet enable them to directly compete with or prey upon native owl species (Franklin et al., 2021). The barred owl's competitive dominance over the spotted owl has been well documented via lethal removal experiments (Diller et al., 2016; Wiens et al., 2021), with spotted owls now at risk of extinction across the Pacific Northwest owing to competitive interactions with barred owls (U.S. Fish and Wildlife Service, 2023). Barred owls also frequently prey on small‐bodied owls such as western screech owls (*Megascops kennicottii*; Baumbusch, 2023), pygmy owls (*Glaucidium californicum*; Baumbusch, 2023), and northern saw‐whet owls (*Aegolius acadicus*; Chick, 2023; Kryshak et al., 2022). When barred owls reach high densities, they can impact the habitat use of western screech owls, leading to declines in detection and occupancy rates in mature forest habitats (Rugg et al., 2023), with potential extirpation of insular populations presumably via predation (Acker, 2012; Elliott, 2006). In areas where barred owls occur at lower density, such as the Sierra Nevada, California, they have comparatively small effects on spotted owls due to reduced niche overlap (Wood et al., 2021), but effects on the broader forest owl assemblage have not been assessed (Hofstadter et al., 2022). Understanding the potential impacts of barred owls on native owl species early in the invasion stage is critical for informing the efforts of federal and state natural resource agencies to manage invasive barred owls, an effort that involves deciding when and where to invest limited management resources (U.S. Fish and Wildlife Service, 2023).

The simultaneous increases in severe wildfire activity and invasive barred owls challenge our ability to assess the relative effects of these two emerging threats on forest owls (Darling & Côté, 2008). Many correlative approaches proposed to diagnose and distinguish causes of endangerment have limitations, including distinguishing impacts from background variability and quantifying the relative—and potentially synergistic—effects of multiple factors (Peery et al., 2004). For example, barred owls may have relatively strong effects on individual forest owls and on populations already stressed by reductions in habitat quality from severe fire. Experimental approaches, where a stressor of interest is manipulated and the response of a target species is monitored in both treatment and control areas, can circumvent these challenges and provide a powerful framework for diagnosing causes of endangerment (Caughley, 1994). However, multiple potential stressors can rarely be manipulated concurrently, particularly at broad spatial scales, which has limited the rigorous application of experimentation in conservation science.

**FIGURE 1:**
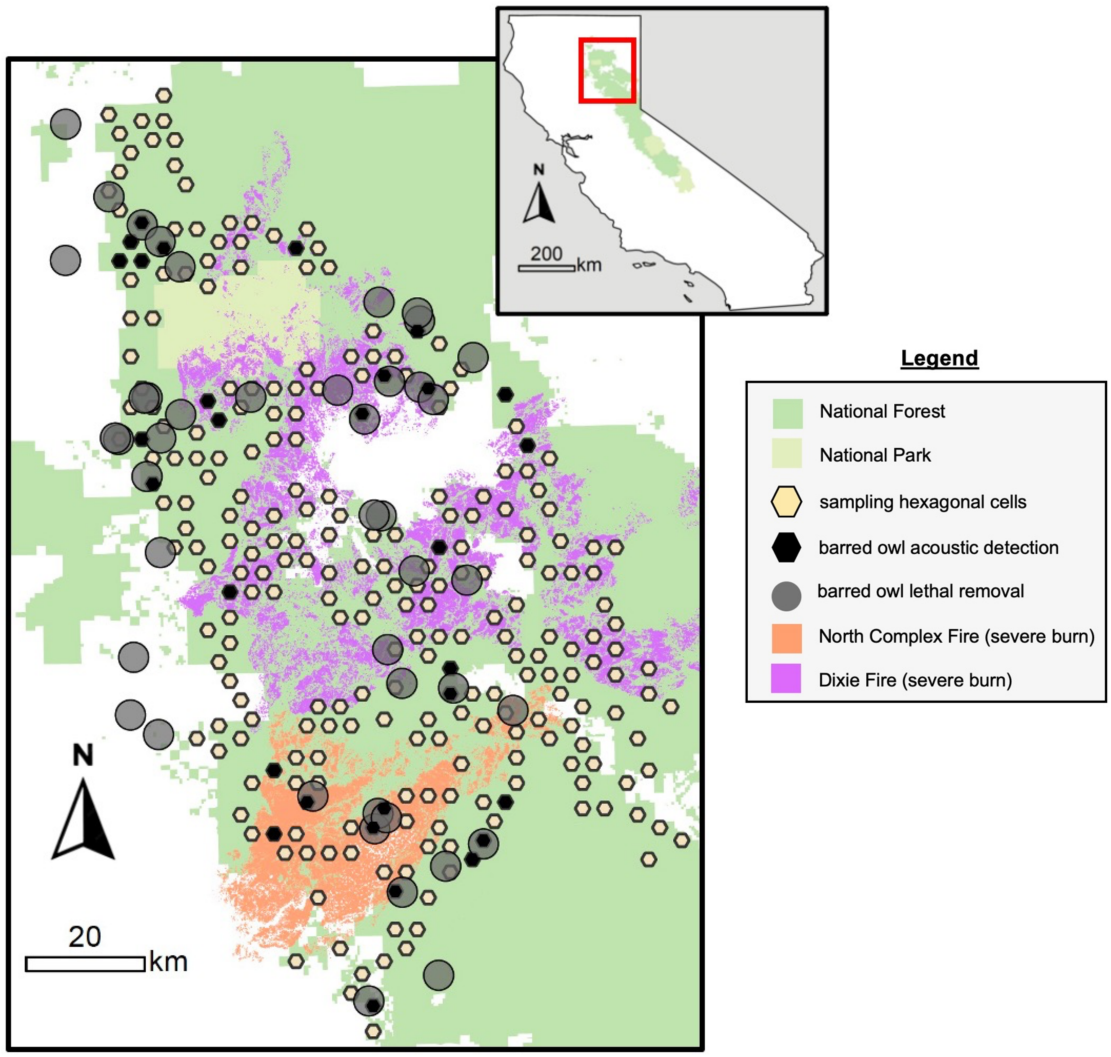
Study area in the northern Sierra Nevada, California, using regional‐scale, passive acoustic surveys from 2018 to 2023. Barred owl acoustic detections are based on 2018 detections before lethal removals.

**FIGURE 2:**
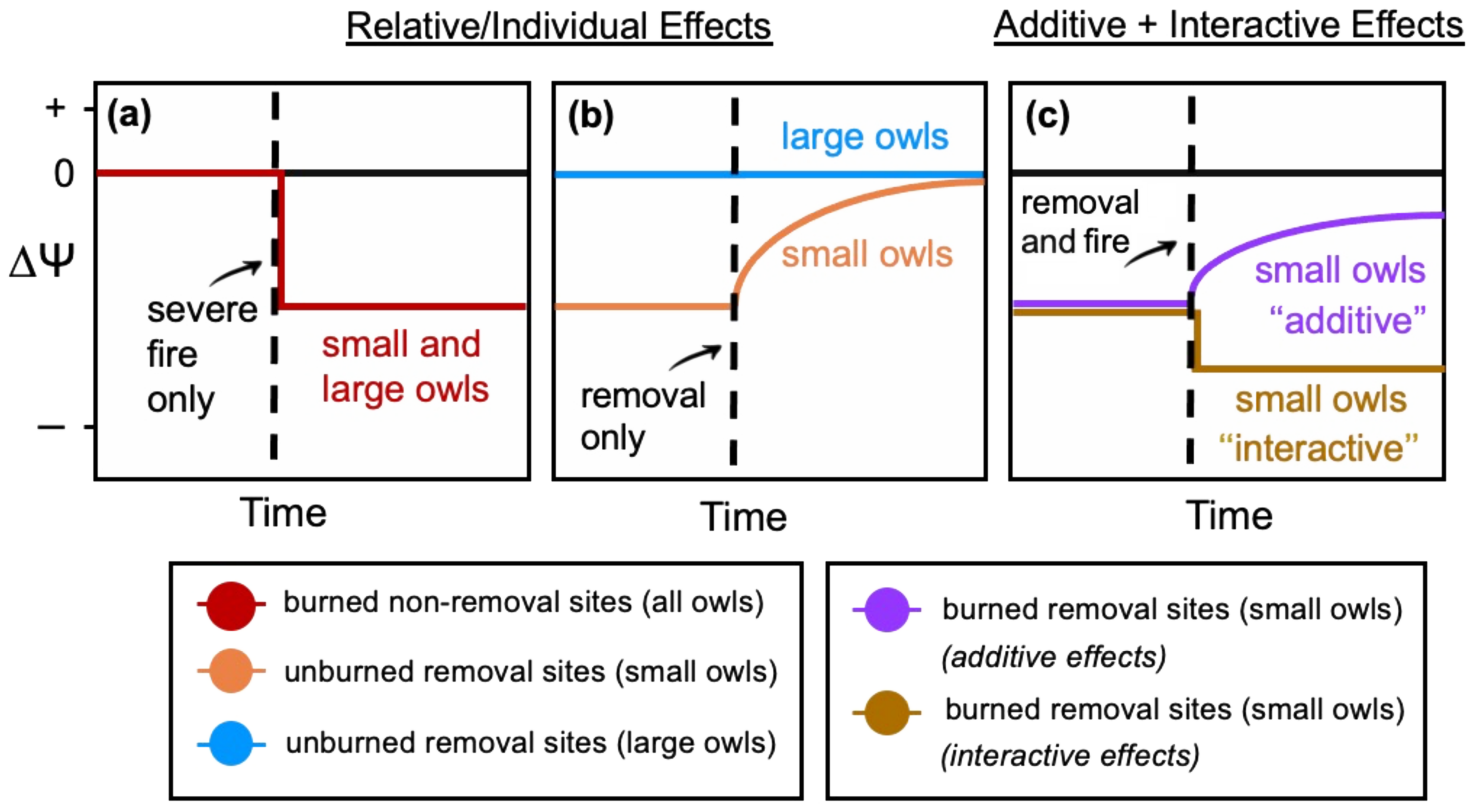
Predicted response of native forest owls in the northern Sierra Nevada region, California, USA, to severe wildfire and barred owl removals, including (a) relative effects of severe wildfire, (b) barred owl lethal removals, and (c) additive and interactive disturbance effects. All plots show predicted changes in forest owl occupancy at impact sites relative to their expected responses at control sites that have not experienced burning or removals (∆Ψ). Native target species included the flammulated (small), pygmy (small), and great horned owl (large). Predictions on the *y*‐axis below 0 indicate that impact sites experience negative change in occupancy rates compared to control sites, whereas predictions above zero suggest that impact sites experience positive change in occupancy rates compared to control sites.

Here, we used a Before‐After Control‐Impact experimental design, leveraging a lethal removal experiment and two severe wildfire events to assess the relative and potentially interactive effects of severe wildfire and invasive barred owls at low densities on a group of native forest owl species across the northern Sierra Nevada region. While lethal removal experiments have been conducted to assess barred owl effects on spotted owls (Diller et al., 2016; Hofstadter et al., 2022; Wiens et al., 2021), ours is the first to use experimentation to test barred owl effects on the broader forest owl community, taking wildfire impacts into account (Duchac et al., 2021). To do so, we conducted regional‐scale, passive acoustic surveys before and after both wildfire and barred owl lethal removal events, collecting data over four breeding seasons (Figure 1). We tested three hypotheses about the effects of these environmental changes on three owl species in western forests. First, we hypothesized that recent large, severe wildfires affected the populations of native owls (possibly by reducing or eliminating structural features or prey densities). Under this hypothesis, we predicted that all three native owl species, pygmy owls, flammulated owls (*Psiloscops flammeolus*), and great horned owls (*Bubo virginianus*), would exhibit decreased occupancy rates at sites that recently experienced high‐severity fire within their home range, and we expected that these lower occupancy rates would persist for several years (Figure 2a).

Second, we hypothesized that barred owls depress populations of smaller bodied owls, possibly via predation. Under this hypothesis, we predicted that smaller bodied owl species (the flammulated owl and pygmy owl) would have higher occupancy rates at lethal removal sites following removals, compared to control sites without removals, owing to the colonization of unoccupied sites post‐removal (Figure 2b). In contrast, we predicted the larger‐bodied species (great horned owl) would exhibit similar patterns in occupancy rates at lethal removal and control sites post‐removal (Figure 2b). Third, we posed two alternative hypotheses about the combined effects of severe fire and the removal of barred owls on smaller bodied owls. Under the hypothesis that the two factors affect smaller bodied owls additively, where severe fire does not inhibit the recolonization of removal sites, we predicted occupancy rates would be relatively low at removal sites that experienced severe fire after removals compared to unburned removal sites but would exhibit similar rates of increase following removals (Figure 2c). Under the hypothesis that the two factors (fire and invader) affect smaller bodied owls interactively, where severe fire limits the recolonization of removal sites, we predicted that smaller bodied owls would only recover at sites that remained unburned following the removal of barred owls (Figure 2c). Finally, we tested for possible niche segregation between native owls and barred owls to explain the potential responses of native owls to barred owls. By adopting a powerful bioacoustics‐based experimental approach, this study can simultaneously assess the impacts of environmental changes, such as habitat loss from severe wildfires and biological invasions, on a regional spatial scale for a group of potentially at‐risk species.

## METHODS

### Study system

Our study area encompassed >6000 km^2^ in the northern Sierra Nevada, California (Figure 1), comprising primarily publicly managed lands (Lassen and Plumas National Forests) and some private lands. The area included a topographically complex mountain range that varied in elevation and vegetation type (though predominantly mixed conifer forest). Three significant environmental changes occurred in this region from 2018 to 2022: (1) the North Complex Fire (1220 km^2^ burned in late summer 2020, 599 km^2^ [49%] at high severity); (2) the Dixie Fire (3740 km^2^ burned in late summer 2021, 2090 km^2^ [56%] at high severity); and (3) a barred owl lethal removal experiment (removals conducted across the entire study area primarily in summer 2019 described below) (Hofstadter et al., 2022). The extent and severity of each fire were both a departure from historical fire regimes and emblematic of emerging trends in the Sierra Nevada (Cova et al., 2023).

The lethal removal experiment was initiated after documentation of rapid population growth in the project area between 2017 and 2018 (Wood, Gutiérrez, et al., 2020); most removals were conducted in 2019 (54 birds), with 8 owls removed in 2018, 14 owls removed in 2020, and 2 in 2021 and 2022 (Hofstadter et al., 2022). We lethally removed barred owls and hybrids using a 12‐ or 20‐gauge shotgun following field protocols established by Diller et al. (2014, 2016), and all removals were carried out by trained and permitted personnel from Sierra Pacific Industries and the University of Wisconsin (Hofstadter et al., 2022). Of the 80 total removals, 67 were barred owls and 13 were barred owl × spotted owl hybrids. We grouped barred owls and hybrids due to their genetic similarity and small hybrid sample size, which prevented a separate analysis for barred owls and hybrids.

Between 2018 and 2020, barred owl site occupancy decreased from 0.19 to 0.03—a reduction factor of 6.3—along with high site extinction rates of 0.92 (Hofstadter et al., 2022), suggesting a significant treatment effect despite having fewer barred owl removals compared to efforts in higher density regions (Wiens et al., 2021). By 2023, barred owl naïve occupancy was 0.006 across the entire Sierra Nevada study region (Winiarski et al., 2024). After major lethal removals in 2020, barred owls were acoustically detected at two sites in 2021 (both single‐night detections, likely of transient individuals), one site in 2022, and two sites in 2023. Hence, we excluded barred owls from occupancy analyses of removal effects due to minimal post‐removal detections. Similarly, we excluded spotted owls from these analyses because their response to barred owl removals has already been published using this dataset. Hofstadter et al. (2022) reported that 56% of previously occupied spotted owl territories were recolonized following barred owl removals, indicating a positive response. Since spotted owls have been extensively studied in relation to both fire and barred owl invasion (e.g., Jones et al., 2016; Kramer et al., 2021; Rockweit et al., 2017), we focused our analyses on other forest owl species for which barred owl and fire impacts remain poorly understood.

### Passive acoustic surveys

To characterize the effects of severe fire and invasive barred owls on forest owl communities, we conducted passive acoustic surveys in the northern Sierra Nevada from early May to late July or the onset of wildfire in 2018, 2021, 2022, and 2023, bracketing the periods in which the North Complex Fire (August to September, 2020) and Dixie Fire (July to October, 2021) burned and barred owls were removed (survey data from 2019 and 2020 was excluded because lethal removals and acoustic surveys occurred simultaneously, violating the assumption of closure). Our study relied on nocturnal surveys from 2000 to 0800 h PDT, aligning with the activity periods of most owls and capturing the crepuscular behavior of pygmy owls (Sater et al., 2006). Although pygmy owls are primarily diurnal hunters, their frequent detections during the crepuscular period justified their inclusion in our analysis (Deshler et al., 2019; Sater et al., 2006). However, because our surveys did not extend into full daylight hours, some pygmy owl detections were likely missed.

Passive acoustic surveys can provide detection/non‐detection data required for occupancy modeling (Wood et al., 2019). Each year we surveyed the same 265 hexagonal cells, each 400 ha, similar in size to spotted owl and barred owl territories in the region (Wood, Gutiérrez, et al., 2020). We systematically selected approximately 1 of 5 hexagonal cells from the grid in the Lassen and Plumas National Forests for surveying, ensuring they were (1) non‐contiguous to minimize nonindependence among sites (e.g., detecting the same owls in multiple hexagonal cells) and (2) readily accessible by road. In each hexagonal cell, we deployed two autonomous recording units (ARUs; Swift One Recorder, K. Lisa Yang Center for Conservation Bioacoustics, Cornell Lab of Ornithology, Ithaca, NY) at locations with high topographic relief and a minimum spacing of 500 m. The elevations of sites surveyed ranged from 669 to 2262 m with a mean of 1573 m. In 2018, we deployed ARUs three times at each site for approximately 1 week per deployment, with an average interval of 26 days (minimum 14 days) between deployments. In 2021, 2022, and 2023, we deployed ARUs continuously for 5 weeks at each site. Although total survey effort was lower and the deployment schedule differed in 2018, all deployments occurred during the core breeding season (May–July) and at the same spatial locations across years. We also accounted for sampling effort differences by including sampling effort as a fixed effect in our detection models (see below). We deployed ARUs in the same locations each year. Equipped with an omnidirectional microphone, the ARUs recorded sound data at a sample rate of 32 kHz, 16‐bit resolution, and gain of +33 dB.

### Processing acoustic survey data

To identify forest owl vocalizations, we used the BirdNET algorithm, a deep convolutional neural network designed to identify >6000 species by sound, most of them birds (Kahl et al., 2021). We used a custom version of BirdNET overfit to vocalizations of the species of interest in this study. Every 3 s, BirdNET produces a unitless numeric prediction score from 0 to 1 for each species. The prediction score reflects its relative confidence in correctly identifying the species, with higher scores representing higher confidence (Wood & Kahl, 2024).

To determine the occurrence of a species‐specific vocalization, we selected minimum prediction score thresholds that would result in false‐positive rates of <1% for each species at the scale of a weekly secondary sampling period. To do so, we first used the program Raven Pro (K. Lisa Yang Center for Conservation Bioacoustics, Cornell Lab of Ornithology) to manually review a random subset of at least 200 h of audio data each year for each species, with all selected hours containing at least one BirdNET prediction with a score ≥0.1. When reviewing the acoustic data, we assigned a code of “1” to an hour‐long sample if it contained at least one confirmed true positive or “0” if it did not contain at least one confirmed true positive. In each hour‐long sample, we then counted the number of BirdNET predictions above a range of scores (0.1, 0.2,…, 0.9; 0.91,…, 0.99). Using logistic regression, we estimated the probability of a false positive in a random hour of acoustic data as a function of the number of BirdNET predictions over each score, where we treated the true positive/false positive status as a binary response (i.e., false positive = 1; true positive = 0) and the number of BirdNET predictions in an hour above a given score threshold as the predictor (lme4; Bates et al., 2015). We estimated false positive rates for each potential threshold at the scale of a secondary sampling period using the equation 1 − (1 − FP)^*n*^, where FP was the hourly false positive rate, and *n* was the number of hours in a typical secondary sampling period (*n* = 84 h; see below). We selected thresholds that yielded an expected <1% false positive rate for each species by year combination.

As a result of transitioning to different microphones between 2018 and 2021 and maintaining the same microphones from 2021 onward, we decided to uniquely assess audio data from the 2018 and 2021 seasons, with call rate thresholds in 2022 and 2023 matching those from 2021. To increase the sample size before and after wildfire and lethal barred owl removals, we manually validated predictions for pygmy owls and flammulated owls with a 15% and 42% false positive rate, respectively (Appendix S1: Table S1). We initially included western screech owls and saw‐whet owls in our analyses, but we had to exclude them due to low detection occurrences, both at barred owl lethal removal and severely burned sites.

### Creating encounter histories for occupancy modeling

For flammulated and pygmy owls, we created encounter histories from the 4 years of passive acoustic survey data at the level of individual ARUs, as these species have relatively small home ranges and quiet calls, meaning ARUs separated by ≥500 m could be treated as independent sampling units. For great horned owls, a larger species with a larger home range and louder calls, we created encounter histories at the level of sampling hexagons (i.e., two ARUs), as multiple ARUs within a 400‐ha hexagon may record calls from the same individual. We divided the survey season into 11 1‐week‐long secondary sampling periods, starting on May 7 and ending on July 23. We assumed population closure throughout the year. If an ARU did not record during a specific secondary sampling period, we treated that period as null. However, if an ARU was active for only part of a secondary sampling period, we included the data from that period and treated survey hours as a detection covariate to account for unequal sampling (see below).

### Environmental predictors

To calculate predictor variables, we created circular buffers around all sites that represent the approximate size of each species' home range (Bennett & Bloom, 2005; Giese & Forsman, 2003; Yanco & Linkhart, 2018). For flammulated and pygmy owls, we buffered individual ARUs at 50 and 300 ha, respectively. To represent larger home ranges in great horned owls, we delineated buffers of 700 ha around the center of the sampled 400‐ha hexagons. Next, we utilized severely burned area (with at least 75% overstory mortality) as estimated by the Monitoring Trends in Burn Severity database (www.mtbs.gov) to generate two wildfire predictors: (1) a binary response of either burned (1) or unburned (0) indicating whether any severe fire occurred within a home range buffer and (2) a continuous value ranging from 0 to 1 representing the proportion of the area that burned at high severity during the Dixie Fire and North Complex Fire, providing a measure of the local extent of severe fire (Appendix S1: Figure S1).

We created a binary predictor to represent the lethal removal of barred owls based on the 57 lethal removal locations of barred owls and barred owl × spotted owl hybrids from 2018 through 2022 that overlapped with our acoustic study area (Hofstadter et al., 2022). We first delineated a circular buffer of 2004 ha around each barred owl removal location, the approximate home range size of this species in the region (Wood, Gutiérrez, et al., 2020). Then, for flammulated and pygmy owls, we classified a site (i.e., ARU) as “1” if their species‐specific home range buffer overlapped with a barred owl home range buffer, and as “0” if no such overlap occurred. For great horned owls, we designated sites as “1” if a great horned owl's home range buffer centered around a hexagon overlapped a barred owl's home range buffer. We coded sites as “0” if the great horned owl's home range buffer did not overlap a barred owl's home range buffer.

### Modeling site occupancy probability

We developed Bayesian single‐species “stacked” single‐season occupancy models to assess the effect of barred owls and wildfire on site occupancy rates for the three forest owl species and controlled for imperfect detection (MacKenzie et al., 2018). We stacked the four‐year encounter histories (2018, 2021, 2022, 2023) because we were more interested in regional owl occupancy patterns rather than turnover rates. We considered a year and site random effect on occupancy (see below) because the stacked model assumes independence among sites and therefore may underestimate error in model coefficients. We also chose this data format to increase the effective sample size, thereby enabling us to fit a larger set of occupancy predictors for less prevalent species and avoid difficulties associated with poor model fit.

We first modeled the potential effects of five predictors as fixed effects on the probability of detecting each species. We considered ordinal date as a predictor to accommodate potential changes in vocalization patterns over the breeding season (Crozier et al., 2003). We also considered the year as a predictor to assess interannual variation in detectability and account for different microphones in 2018. We included terrain ruggedness as a predictor because owl vocalizations may travel a shorter distance in areas of complex topography. We calculated terrain ruggedness by averaging the absolute differences between the elevation of a 10‐m raster grid cell and the values of its eight surrounding cells within a 390‐m buffer around each ARU location, where elevation data were obtained from a digital elevation model (Hijmans et al., 2015). We treated the number of hours ARUs recorded per secondary sampling period as a predictor, given that units were deployed for variable durations. We used the barred owl detection/non‐detection history obtained from our passive acoustic surveys as a predictor to account for potential changes in vocalization behavior in response to barred owl presence (Rugg et al., 2023; Wood, Klinck, et al., 2020). We coded hexagonal cells as “1” if a barred owl was detected at least once in a given year at either ARU or “0” if no detection occurred during that year. In 2018, barred owls were acoustically detected at 31 hexagonal cells, 18 of which overlapped with follow‐up barred owl lethal removal locations. We did not include background noise as a detection covariate to maintain a minimal set of covariates because BirdNET is robust to high ambient noise (Kahl et al., 2021). No pairs of predictors were highly correlated (all Pearson's correlation coefficients <0.6). We incorporated all five predictors into the occupancy models for each species to minimize the potential number of models (see below).

Next, we built six individual occupancy models to assess whether barred owl removals, severe wildfires, or interactions between the two predictors affected owl occupancy rates. We treated unburned and barred owl non‐removal sites as control sites for each owl species and employed an unbalanced Before‐After‐Control‐Impact design.

To test for severe fire effects independent of time since burn, we created the *Fire Time‐Constant Model*:

(1) $$\text{logit}\left(\Psi_{i}\right)=\beta_{0}+\beta_{1}{\text{burn}}_{i}+\beta_{2}{\text{allpostburn}}_{i}+\mu_{1}\left[1|{\text{year}}_{t}\right]+\mu_{2}\left[1|{\text{site}}_{i}\right]$$

where burn_*i*_ was an indicator predictor for burned sites (i.e., burned = 1; unburned = 0), allpostburn_*i*_ was a continuous predictor representing the proportion of the area that burned at high severity at any point postfire, year_*t*_ was a random effect indicator for survey year, and site_*i*_ was a random effect for individual sites (i.e., ARUs for flammulated and pygmy owls and hexagonal cells for great horned owls).

To test for severe fire effects while accounting for different years postburn, we created the *Fire Time‐Dependent Model*:

(2) $$\text{logit}\left(\Psi_{i}\right)=\beta_{0}+\beta_{1}{\text{burn}}_{i}+\beta_{2}\text{burn}1_{i}+\beta_{3}\text{burn}2_{i}+\beta_{4}\text{burn}3_{i}+\mu_{1}\left[1|{\text{year}}_{t}\right]+\mu_{2}\left[1|{\text{site}}_{i}\right]$$

where burn_*i*_ was the same as in the *Fire Time‐Constant Model* and burn1_*i*_, burn2_*i*_, and burn3_*i*_ were continuous predictors representing the proportion of the area that burned at high severity relative to the number of years postfire (i.e., all prefire sites and unburned sites postfire = 0).

To test for barred owl effects independent of time since removal, we developed the *Barred Owl Time‐Constant Model*:

(3) $$\text{logit}\left(\Psi_{i}\right)=\beta_{0}+\beta_{1}\text{site}‐{\text{type}}_{i}+\beta_{2}{\text{allpostlethal}}_{i}+\mu_{1}\left[1|{\text{year}}_{t}\right]+\mu_{2}\left[1|{\text{site}}_{i}\right]$$

where site‐type_*i*_ was an indicator predictor for barred owl removal sites (i.e., removals = 1; no removals = 0 based on the criteria described above), allpostlethal_*i*_ was an indicator predictor for removal sites at any point post‐removal, year_*t*_ was a random effect indicator for survey year, and site_*i*_ was a random effect for individual sites (i.e., ARUs for flammulated and pygmy owls and hexagonal cells for great horned owls).

To test for barred owl effects while accounting for years since removal, we built the *Barred Owl Time‐Dependent Model*:

(4) $$\text{logit}\left(\Psi_{i}\right)=\beta_{0}+\beta_{1}\text{site}‐{\text{type}}_{i}+\beta_{2}\text{lethal}1_{i}+\beta_{3}\text{lethal}2_{i}+\beta_{4}\text{lethal}{3^{+}}_{i}+\mu_{1}\left[1|{\text{year}}_{t}\right]+\mu_{2}\left[1|{\text{site}}_{i}\right]$$

where site‐type_*i*_ was the same as in *Barred Owl Time‐Constant Model* and lethal1_*i*_, lethal2_*i*_, and lethal3^+^_*i*_ were indicator predictors for removal sites one, two, and three or more years post‐removal (i.e., removal sites post‐removal = 1; all pre‐removal sites and non‐removal sites post‐removal = 0). We evaluated for the effects of barred owl lethal removal parameters by comparing native owl occupancy at removal sites to occupancy at those same sites in the years following removal. Since lethal removals happened in different years, a direct before‐and‐after comparison between control and impact sites was not possible. To address this, we used a contrast analysis to measure overlap between posterior distributions at removal sites (i.e., site‐type_lethal_) and post‐removal sites (i.e., lethal1_lethal_, lethal2_lethal_, lethal3^+^_lethal_, and allpostlethal_lethal_) (Pastore & Calcagnì, 2019). We considered differences between posterior distributions to be biologically meaningful if their overlap was less than 15% (Appendix S1: Figure S2).

To test for interactions between fire and barred owl removals independent of time since burn, we created the *Interaction Time‐Constant Model*:

(5) $$\text{logit}\left(\Psi_{i}\right)=\beta_{0}+\beta_{1}\text{site}‐{\text{type}}_{i}+\beta_{2}{\text{burn}}_{i}+\beta_{3}{\text{allpostlethal}}_{i}\times {\text{burnYrs}}_{i}+\mu_{1}\left[1|{\text{year}}_{t}\right]+\mu_{2}\left[1|{\text{site}}_{i}\right]$$

where site‐type_*i*_ and burn_*i*_ were indicator predictors for barred owl removal and burned sites (i.e., removal or burned site = 1; no removals or unburned site = 0 based on the criteria described above) and allpostlethal_*i*_ × burnYrs_*i*_ indicated sites where barred owl removals preceded wildfire, measured for all years postfire (i.e., burned removal site postburn = 1; all other sites = 0).

Finally, to test for interactions between fire and barred owl removals while accounting for time since burn, we developed the *Interaction Time‐Dependent Model*:
(6)

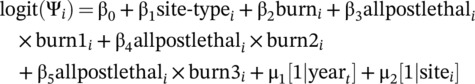

>where site‐type_*i*_ and burn_*i*_ were the same as in *Interaction Time‐Constant Model* and allpostlethal_*i*_ × burn1_*i*_, allpostlethal_*i*_ × burn2_*i*_, and allpostlethal_*i*_ × burn3_*i*_ were indicator predictors for sites with interactions between barred owl removals and severe fire depending on the years since the fire occurred (i.e., allpostlethal_*i*_ × burn3_*i*_ = 1 indicates a site where a barred owl removal was followed by high‐severity fire in the third year after the fire, with the covariate representing a binary indicator rather than a continuous fire severity measure).

To evaluate the magnitude and certainty of the beta coefficient estimates in all models, we assessed whether their 85% credible intervals overlapped with zero and examined the proportion of the posterior distribution that is in the same direction as the mean estimate. To estimate the marginal effects of predictors on both detection and occupancy within a Bayesian framework, we ran four chains of 2600 iterations and discarded the initial half of iterations as burn‐in. We then employed a MacKenzie–Bailey goodness‐of‐fit test (posterior predictive checks) to evaluate model fit (MacKenzie & Bailey, 2004) (see Appendix S1: Figure S3). We evaluated model convergence through trace plots and Gelman‐Rubin statistics ($\hat{r}\leq 1.1$; Gelman & Rubin, 1992) (see Appendix S1: Figure S4). We performed occupancy analyses using the R package “ubms” (Kellner et al., 2022) and used its predict function to estimate and visualize occupancy probabilities.

### Testing for habitat niche segregation

We fit Bayesian single‐species, single‐season occupancy models to examine potential habitat niche segregation between native owl species and barred owls. Our focus was on a specific subset of niche components that could help elucidate the findings of the occupancy analysis or be pertinent to management strategies. For the native species models, we exclusively used confirmed detections from the 2018 passive acoustic surveys, capturing conditions after barred owl establishment but before removals and wildfire. These data may therefore reflect habitat preferences under early invasion pressure, representing pre‐removal conditions. For barred owl models, we used confirmed detections of barred owls from the 2018 passive acoustic surveys and locations where barred owls were lethally removed between 2018 and 2020, preceding both wildfire incidents. In our barred owl models, we fit a logistic regression using the R package *stats* (R Core Team, 2022) because our barred owl occurrence data incorporated nonrepeat surveying methods and barred owls exhibit high seasonal detection probabilities in the region, with a detection probability of 0.89 across two visits (Wood et al., 2019). For both native owls and barred owl models, we included terrain ruggedness as a predictor because owl species can use areas characterized by a range of topographic features (Linkhart et al., 1998), calculating ruggedness as described above. We also treated elevation as a predictor because some owls may occupy habitats at distinct elevations based on resource needs or changing seasonal temperatures (Duchac et al., 2021). Next, we treated intermediate‐to‐late seral forests as a predictor because forest structure often influences owl presence. We calculated intermediate‐to‐late seral forests as the proportion of home range area where dominant trees have medium and large diameters (as determined by 2017 GNN; QMD ≥ 25 cm) and canopy cover greater than 40% (Kramer et al., 2021). No pairs of predictors were highly correlated (all Pearson's correlation coefficients <0.6). Finally, we analyzed niche breadth and niche overlap between native owls and barred owls using the R packages *ellipse* and *sf* (Murdoch & Chow, 2023; Pebesma & Bivand, 2023). We determined niche breadth by evaluating habitat utilization frequency in the study area, with 85% standard ellipses depicting environmental conditions used by owls based on their detection records.

## RESULTS

We deployed 517 ARUs across 265 hexagonal cells in 2018 and 2021, and 477 and 401 ARUs in 256 and 214 hexagonal cells, respectively, in 2022 and 2023. We collected 84,320 h of acoustic audio data in 2018, 217,908 h in 2021, 230,112 h in 2022, and 172,596 h in 2023. From 2018 to 2023, naïve occupancy for flammulated owls ranged between 0.17 and 0.30; for pygmy owls it ranged from 0.10 to 0.22; and for great horned owls it ranged from 0.16 to 0.35 (Appendix S1: Figure S5, Table S1).

### Owl occupancy in response to wildfire and barred owl lethal removals

#### Owl detection probabilities

Detection probabilities varied with ordinal date, terrain ruggedness, and weekly survey effort for all species; the presence of barred owl detections influenced the detection of flammulated—but not pygmy owls or great horned owls. Detection probabilities were highest for each species before barred owl removals and both wildfires, although the marginal effect of year on owl detection probabilities was relatively low. Flammulated owl detection declined with the ordinal date (β_OrdinalDate_ = −1.30; 85% CI: [−1.56, −1.04]) and increased with terrain ruggedness (β_ruggedness_ = 0.28; 85% CI: [0.20, 0.36]), weekly survey hours (β_surveyhrs_ = 0.57; 85% CI: [0.45, 0.68]), and barred owl presence (β_BOdet_ = 0.34; 85% CI: [−0.11, 0.81]). Detection probability for pygmy owls was generally lower than flammulated owls but followed similar trends with the ordinal date (β_OrdinalDate_ = −1.93; 85% CI: [−2.29, −1.60]), terrain ruggedness (β_ruggedness_ = 0.22; 85% CI: [0.12, 0.32]), and weekly survey hours (β_surveyhrs_ = 0.84; 85% CI: [0.68, 1.00]). Pygmy owl detection probabilities did not vary with barred owl presence (β_BOdet_ = −0.08; 85% CI: [−0.59, 0.42]), as the marginal effect of barred owl presence on pygmy owl detections was low. Great horned owl detection declined as the ordinal date advanced (β_OrdinalDate_ = −1.18; 85% CI: [−1.57, −0.79]), decreased with terrain ruggedness (β_ruggedness_ = −0.51; 85% CI: [−0.66, −0.37]), and increased with weekly survey hours (β_surveyhrs_ = 1.12; 85% CI: [0.92, 1.31]). Great horned owl detection probabilities did not vary with barred owl presence (β_BOdet_ = −0.33; 85% CI: [−0.88, 0.21]), as the marginal effect of barred owl presence on great horned owl detections was low.

#### Flammulated owl occupancy rates

**FIGURE 3:**
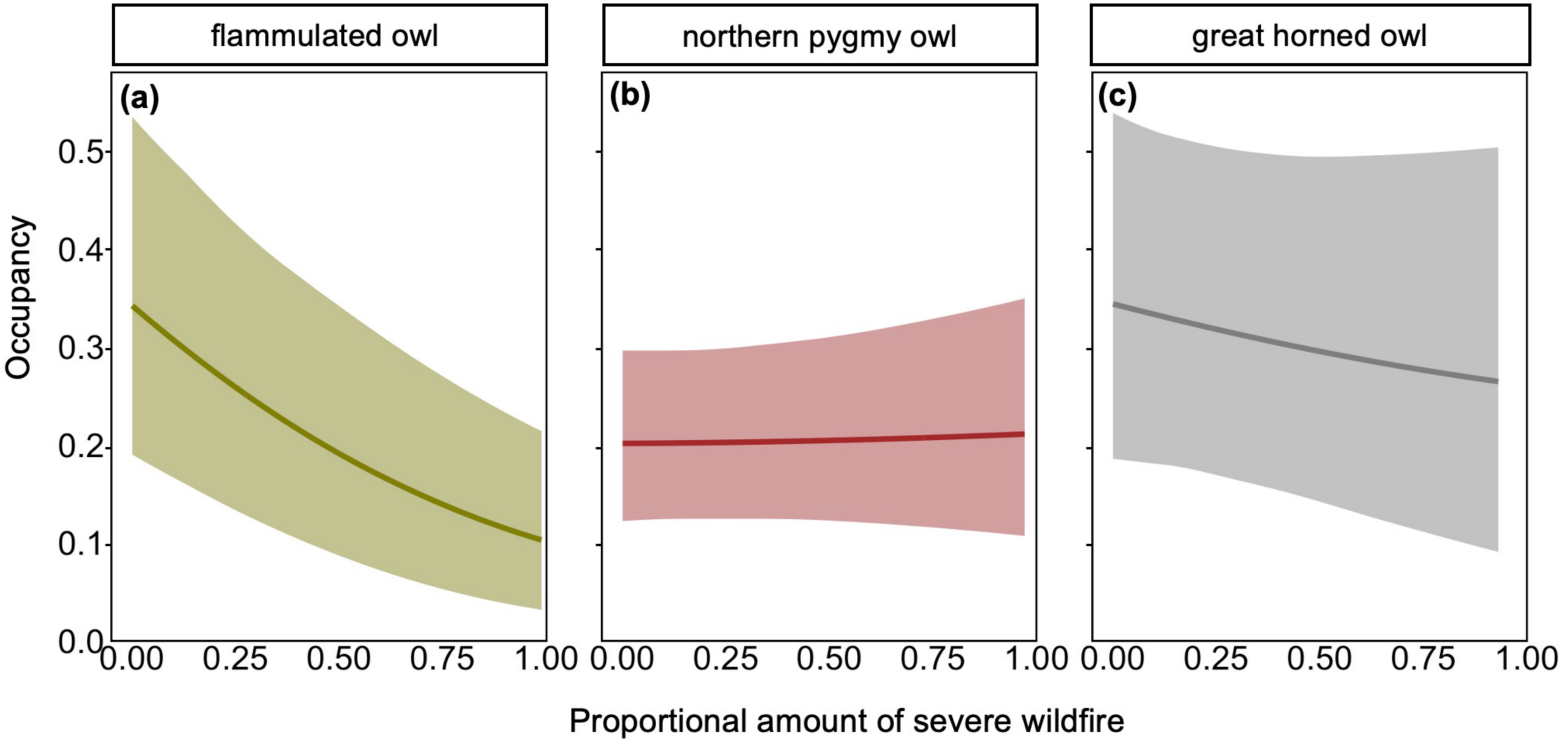
Estimated site occupancy for (a) flammulated owls, (b) pygmy owls, and (c) great horned owls in response to severe wildfire with 85% credible intervals.

**FIGURE 4:**
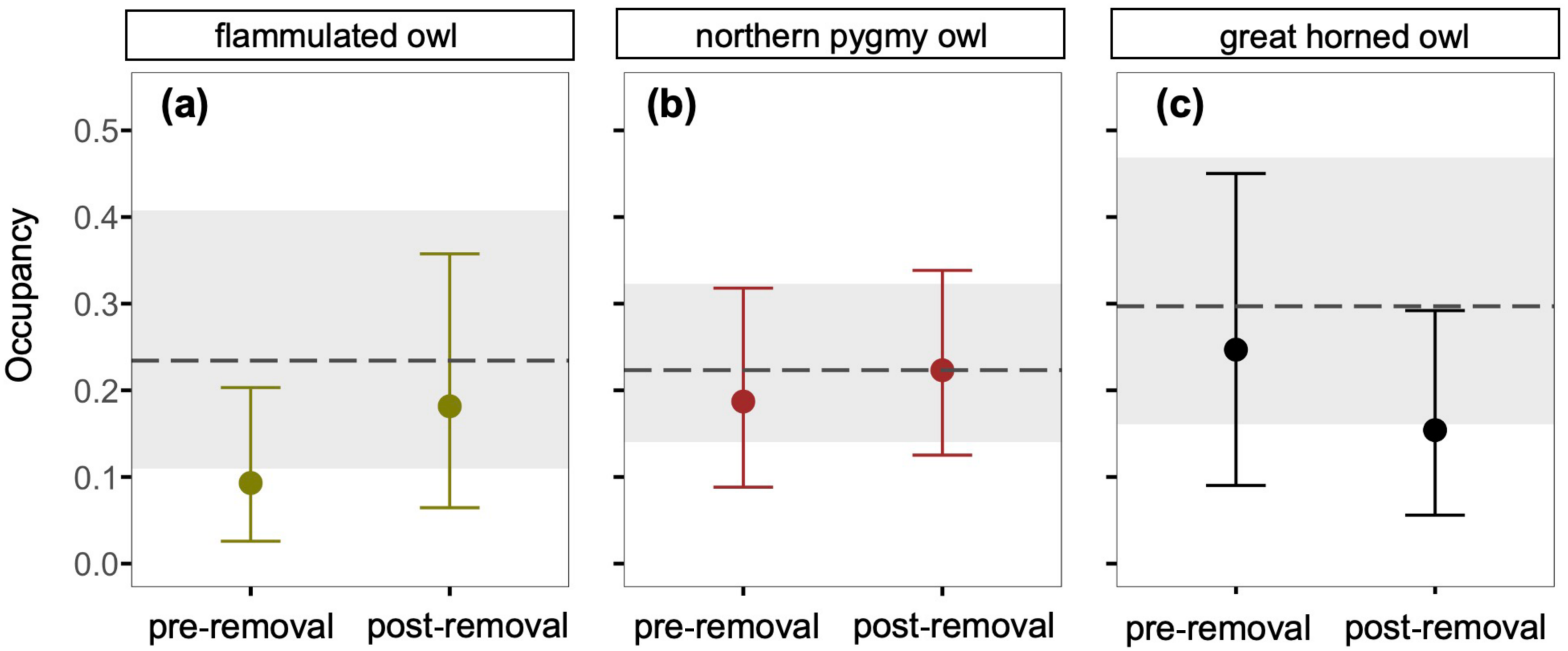
Estimated site occupancy for (a) flammulated owls, (b) pygmy owls, and (c) great horned owls at control sites, removal sites before lethal barred owl removals, and removal sites all years after lethal barred owl removals in the northern Sierra Nevada, California. In each panel, the two bullets represent mean occupancy estimates for the native species at removal sites, shown with 85% credible intervals. The horizontal dashed line marks the mean estimated occupancy at control sites, and the gray shaded band represents the corresponding 85% credible interval. A strict before‐and‐after comparison at control sites was not feasible because lethal removals occurred in different years across the study area.

In the *Fire Time‐Constant Model*, flammulated owls were more likely to occupy sites where some high‐severity wildfire occurred (i.e., % severe wildfire is >0) (Ψ = 0.29; 85% CI: [0.15, 0.47]) compared to unburned sites (Ψ = 0.16; 85% CI: [0.08, 0.29]). However, the *Fire Time‐Constant Model* indicated that wildfires reduced flammulated owl site occupancy by 71% in severely burned areas (sites experiencing near‐complete high‐severity fire) for at least 3 years postfire (Figure 3a). In the *Fire Time‐Dependent Model*, flammulated owls showed consistently negative declines in occupancy postburn across years (Appendix S1: Figure S6). Posterior estimates indicated a 0.93 to 0.99 probability that severe fire would negatively impact flammulated owl occupancy (Appendix S1: Table S2). Next, in the *Barred Owl Time‐Constant Model*, flammulated owls were less likely to occupy removal sites before barred owl lethal removals (Ψ = 0.09; 85% CI: [0.03, 0.20]) compared to control sites without barred owl lethal removals (Ψ = 0.23; 85% CI: [0.11, 0.41]) (Figure 4a). However, this model indicated that flammulated owl occupancy increased at lethal removal sites following removals (Ψ = 0.18; 85% CI: [0.07, 0.36]) (Figure 4a), with only 4% overlap in the posterior distributions on the logit scale (Appendix S1: Table S3, Figure S2). In the *Barred Owl Time‐Dependent Model*, flammulated owls showed variable yet consistently positive occupancy rates at removal sites following removals over time (Appendix S1: Figure S7). Last, both of our interaction models indicated the absence of any interactive effect between severe wildfires and barred owl lethal removals on flammulated owl occupancy.

#### Pygmy owl occupancy rates

In the *Fire Time‐Constant Model*, pygmy owls were less likely to occupy sites where some high‐severity wildfire occurred (i.e., % severe wildfire is >0) (Ψ = 0.20; 85% CI: [0.12, 0.30]) compared to unburned sites (Ψ = 0.24; 85% CI: [0.15, 0.35]), but there was no support for postfire effects in either fire model (Figure 3b). Next, in the *Barred Owl Time‐Constant Model*, pygmy owls were less likely to occupy removal sites before barred owl lethal removals (Ψ = 0.19; 85% CI: [0.09, 0.32]) compared to control sites without barred owl lethal removals (Ψ = 0.22; 85% CI: [0.14, 0.32]) (Figure 4b), but there was no support for post‐removal effects in either barred owl model (Appendix S1: Figure S7). Last, both of our interaction models indicated the absence of any interactive effect between severe wildfires and barred owl lethal removals on pygmy owl occupancy.

#### Great horned owl occupancy rates

In the *Fire Time‐Constant Model*, great horned owls were more likely to occupy sites where some high‐severity wildfire occurred (i.e., % severe wildfire is >0) (Ψ = 0.33; 85% CI: [0.17, 0.52]) compared to unburned sites (Ψ = 0.23; 85% CI: [0.11, 0.39]), but there was no support for postfire effects in either fire model (Figure 3c). Next, in the *Barred Owl Time‐Constant Model*, great horned owls were less likely to occupy removal sites before barred owl lethal removals (Ψ = 0.25; 85% CI: [0.09, 0.45]) compared to non‐removal sites (Ψ = 0.30; 85% CI: [0.16, 0.47]), but there was little support for post‐lethal removal effects in either barred owl model. Last, both of our interaction models indicated the absence of any interactive effect between severe wildfires and barred owl lethal removals on great horned owl occupancy.

### Habitat niche segregation

**FIGURE 5:**
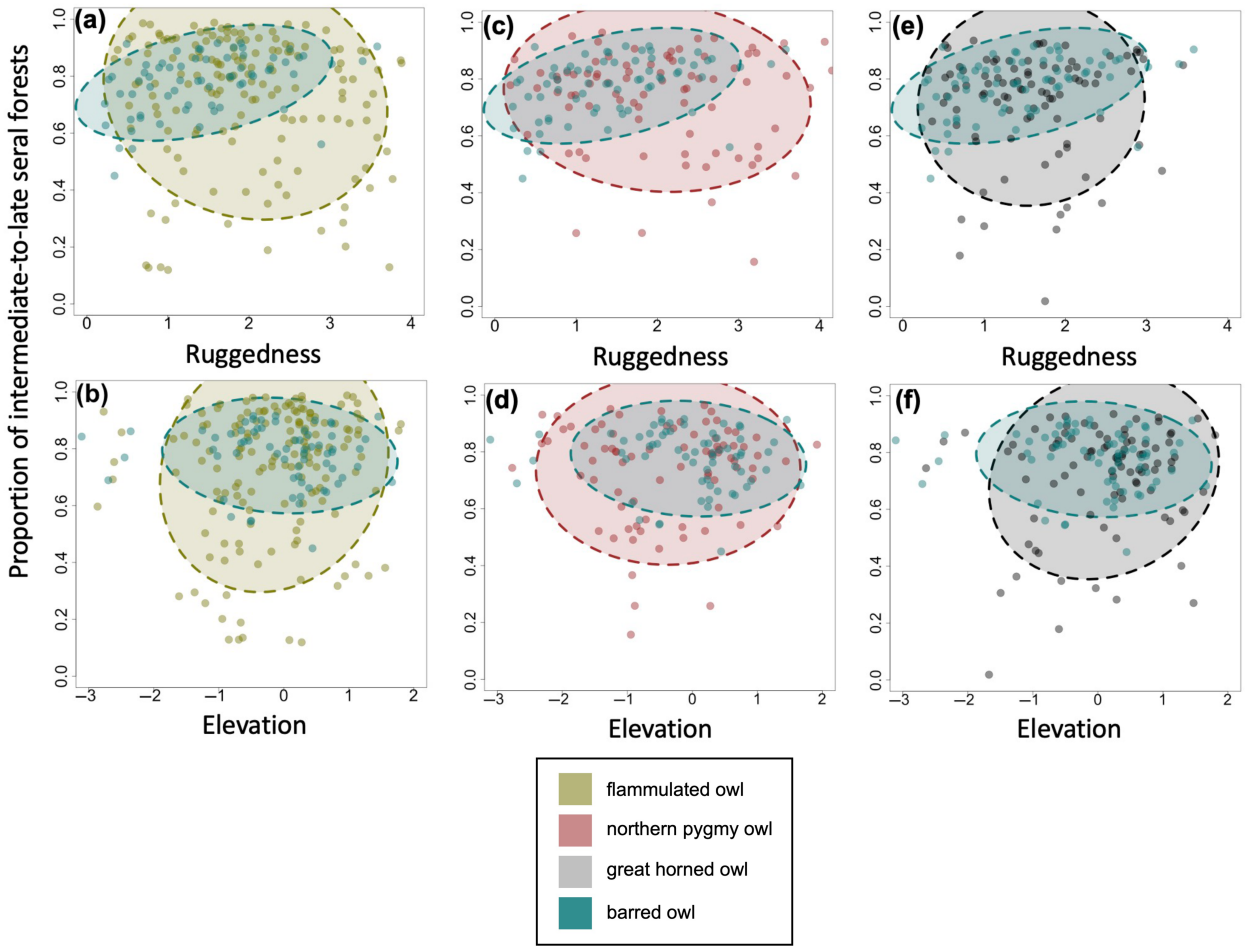
Comparison of barred owl and native forest owl detections at sites relative to elevation, proportion of intermediate‐to‐late seral forest, and terrain ruggedness. Species include barred owls, (a, b) flammulated owls, (c, d) pygmy owls, and (e, f) great horned owls. Elevation was standardized ranging from −3 to 2, with higher values representing higher elevation. Ruggedness ranged from 0 to 4, with higher values representing higher ruggedness. Ellipses encapsulate 85% of the data points and individual dots represent individual sites. For the native species models, we used confirmed detections from the 2018 passive acoustic surveys. For barred owl models, we used confirmed detections of barred owls from the 2018 passive acoustic surveys and locations where barred owls were lethally removed between 2018 and 2020.

**FIGURE 6:**
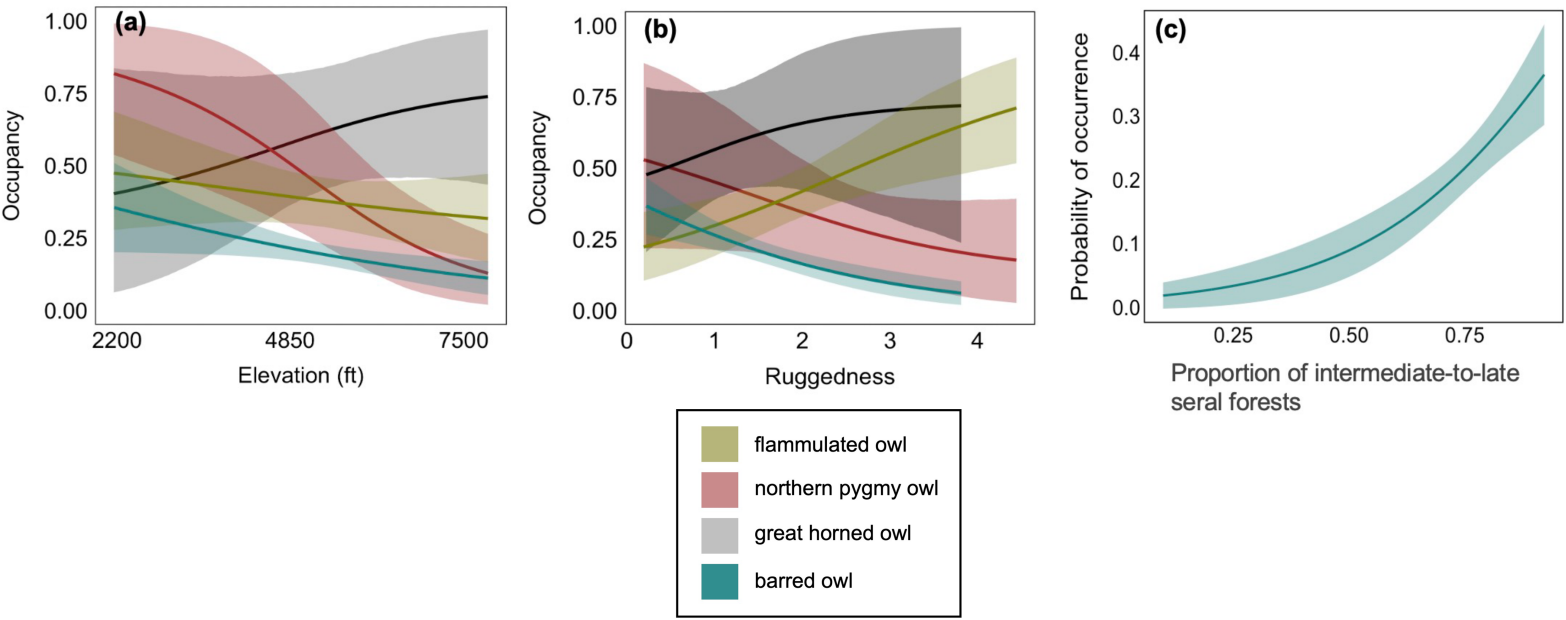
Forest owl occupancy (with 85% credible intervals) relative to elevation, terrain ruggedness, and intermediate‐to‐late seral forests: (a) owl occupancy rates relative to elevation, based on prefire detection data, (b) owl occupancy rates relative to increases in terrain ruggedness, arranged from left to right, and (c) barred owl occurrence with intermediate‐to‐late seral forests. Species include barred owls, flammulated owls, pygmy owls, and great horned owls. For the native species models, we used confirmed detections from the 2018 passive acoustic surveys. For barred owl models, we used confirmed detections of barred owls from the 2018 passive acoustic surveys and locations where barred owls were lethally removed between 2018 and 2020.

Among all owl species, barred owls exhibited the narrowest niche breadth, while pygmy owls and flammulated owls displayed the widest; additionally, flammulated owls showed the least niche overlap with barred owls, followed by pygmy owls and great horned owls (Figure 5; Appendix S1: Table S4). Flammulated owls were more likely to occur at sites at lower elevations (β_elevation_ = −0.13; 85% CI: [−0.41, 0.13]; Figure 6a), with rougher terrain (β_ruggedness_ = 0.55; 85% CI: [0.20, 0.95]; Figure 6b), and less intermediate‐to‐late seral forests (β_intermediate‐to‐late seral forests_ = −0.44; 85% CI: [−1.42, 0.51]), though the credible intervals of elevation and forest size overlapped zero. Flammulated owl site occupancy increased by 196% in the most versus the least rugged areas, in contrast to barred owls, which declined by 84% under similar conditions. Pygmy owls were more likely to occur at sites at lower elevations (β_elevation_ = −0.81; 85% CI: [−1.49, −0.30]; Figure 6a), with less rugged terrain (β_ruggedness_ = −0.49; 85% CI: [−1.16, 0.10]; Figure 6b), and more intermediate‐to‐late seral forests (β_intermediate‐to‐late seral forests_ = 0.38; 85% CI: [−1.00, 1.78]), though the credible intervals of ruggedness and forest size overlapped zero. Great horned owls were more likely to occur at sites at higher elevations (β_elevation_ = 0.36; 85% CI: [−0.25, 1.05]; Figure 6a), with rougher terrain (β_ruggedness_ = 0.50; 85% CI: [−0.56, 1.70]; Figure 6b), and less intermediate‐to‐late seral forests (β_intermediate‐to‐late seral forests_ = −0.45; 85% CI: [−2.15, 1.19]). However, all credible intervals overlapped with zero, and elevation appeared not to significantly differ between sites with great horned owl detections and barred owl removals or detections (Appendix S1: Figure S8). Barred owls were more likely to occur at sites at lower elevations (β_elevation_ = −0.28; 85% CI: [−0.49, −0.06]; Figure 6a), with less rugged terrain (β_ruggedness_ = −0.61; 85% CI: [−0.91, −0.32]; Figure 6b), and greater amounts of intermediate‐to‐late seral forests (β_intermediate‐to‐late seral forests_ = 4.21; 85% CI: [2.56, 6.05]; Figures 5 and 6c). Intermediate‐to‐late seral forests had the greatest average marginal effect (AME) on the likelihood of barred owl occurrence (AME = 0.67; Appendix S1: Figure S9), followed by ruggedness (AME = −0.10) and elevation (AME = −0.04).

## DISCUSSION

Biological invasions and changing disturbance regimes are transforming ecosystems worldwide, casting uncertainty about their future. In forests of western North America, invasive barred owls and rapid forest changes driven by shifting wildfire regimes could profoundly impact biodiversity (Holm et al., 2016; Kryshak et al., 2022; Rizzo & Garbelotto, 2003; Stephens et al., 2018). Our investigation suggests that the impacts of these stressors on forest owls are likely species‐specific, shaped by differences in life history traits such as body size, habitat selection, and diel activity patterns. While severe wildfire had little measurable short‐term impact on great horned owls and pygmy owls, flammulated owls showed a strong negative response to extensive high‐severity burn areas, likely because their small home ranges and insectivorous diet require the close proximity of mature forest foraging habitat and secondary‐cavity nest sites (Peery, 2000; Yanco & Linkhart, 2018), both of which are reduced or eliminated by large, severe fires. Similarly, only flammulated owls showed a positive response to experimental barred owl lethal removals, suggesting that barred owls—despite their relatively low densities and habitat segregation with flammulated owls—may have exerted nonconsumptive or predatory pressure on this nocturnal insectivore. The life history traits of great horned owls, including their larger body size and predatory behavior, along with the partially diurnal activity of pygmy owls, may have buffered these species from barred owl impacts. Unlike previous studies examining these stressors in isolation on avian predators (Duchac et al., 2021; Hannah et al., 2023; Rugg et al., 2023; Weldy et al., 2023), ours evaluates both stressors within an experimental framework. We demonstrate novel insights into the relative and combined impacts of these two major disturbances on a group of forest owls and propose management strategies to conserve species diversity in a changing landscape.

### Effects of severe wildfire on three forest owls

Species respond differently to large, severe wildfires, which can create habitat for species adapted to open habitats but eliminate habitat for forest‐dependent species (Blakey et al., 2020; Jones et al., 2016; Steel et al., 2019, 2021; Wood et al., 2024). In our study, flammulated owl occupancy declined following high‐severity fires, and lower occupancy rates persisted for at least 3 years post‐disturbance, the longest period assessed. The flammulated owl is found in dry mixed conifer forests (Reynolds & Linkhart, 1992), nests in cavities in snags excavated by woodpeckers, and gleans insects from the foliage of large, live conifers (Bull et al., 1990; Linkhart & Reynolds, 1994). Mixed‐severity wildfires promote landscape heterogeneity, creating nesting and foraging opportunities for cavity‐nesting species such as sapsuckers (*Sphyrapicus*) and northern flickers (*Colaptes auratus*) (Hutto & Patterson, 2016; Smucker et al., 2005). Also, severely burned patches can provide nesting opportunities for primary cavity‐nesting species like black‐backed woodpeckers (*Picoides arcticus*), which feed on beetles that colonize burned trees (Dole et al., 2023). Following the Hayman fire in Colorado, flammulated owls eventually recolonized burned areas with less high‐severity fire; however, flammulated owl home ranges were larger where there was more high‐severity fire, likely reflecting the need to travel farther to access suitable foraging habitat (Yanco & Linkhart, 2018). When mixed‐severity fires create small severely burned patches, juxtaposed foraging and nesting habitats may ultimately support flammulated owl persistence (McGinn et al., 2025). In contrast, large, continuous patches of severely burned forest may eliminate insect‐rich foraging habitat and suitable nest cavities in close proximity, contributing to the observed declines in flammulated owl occupancy. As a small‐bodied insectivorous species with necessarily small home ranges (Peery, 2000), flammulated owls may be particularly sensitive to the spatial arrangement of postfire habitat elements.

The longer‐term effects of severe fire on secondary‐cavity‐nesting species like flammulated owls are uncertain. Woodpeckers commonly return to severely burned areas within a few years and create cavities in snags that, along with forest regeneration, improve habitat quality for secondary‐cavity‐nesting species (Swengel, 2001; Tarbill et al., 2015). Snags can remain standing for more than a decade on burned landscapes, especially in sites that are not salvaged‐logged (Russell et al., 2006). However, additional research is needed to understand how fire severity and recovery dynamics impact the long‐term recovery of flammulated owls following high‐severity wildfires.

We found no evidence that severe wildfires reduced great horned owl site occupancy up to 3 years postfire, contrasting with previous studies showing declines for two to 4 years following severe wildfires in the Pacific Northwest and the entire Sierra Nevada (Duchac et al., 2021; McGinn et al., 2025). The great horned owl's resilience in the northern Sierra Nevada, our study area, may stem from the owls' use of open, fragmented landscapes in seasonally dry habitats (Bennett & Bloom, 2005; Ganey et al., 1997) and their diverse diet of rodents, lagomorphs, birds, and other open‐forest species (Kopij, 2016; Marti & Kochert, 1996). While severe wildfire may eliminate large areas of live trees important for great horned owl nesting and roosting, regenerating shrub layers support numerous prey species, with burned forests supporting potentially greater small mammal species evenness (Roberts et al., 2015). Thus, any negative effects of severe wildfire on great horned owls may be outweighed by improved prey accessibility, contributing to this species' apparent resilience to the severe wildfires in our study.

Large areas of severely burned forest also did not reduce pygmy owl site occupancy for at least 3 years postfire. The response of this species to high‐severity fire appears to vary between regions. Previous studies have shown that high‐severity wildfire can limit pygmy owl distribution across the Sierra Nevada 1 year postfire (McGinn et al., 2025) but promote their distribution in the Pacific Northwest 2 years postfire (Duchac et al., 2021). Pygmy owls, like flammulated owls, are secondary‐cavity nesters but have more diverse diets and larger home ranges. Pygmy owls may be adapted to burned landscapes, nesting in snag cavities excavated by woodpeckers postfire and adjusting their foraging behaviors to prey on taxa that persist in burned landscapes (Giese & Forsman, 2003; Holt & Leroux, 1996). In a broader regional study across the Sierra Nevada, pygmy owls were more likely to occur in sites with patchier high‐severity burns interspersed with unburned forest (McGinn et al., 2025). While severe wildfires can reduce site occupancy for some owls in the short term, they may promote habitat for these owls over time, as well as create conditions that enhance foraging and/or nesting habitat for other owl species in the near term.

### Effects of invasive barred owls on three forest owls

Five years of monitoring following the initiation of experimental removals revealed that flammulated owl occupancy increased twofold from pre‐ to post‐removal conditions in response to barred owl removals. Because flammulated owls are largely insectivorous (Linkhart & McCallum, 2013) and barred owls consume mostly vertebrate prey by biomass (Kryshak et al., 2022), the increase in flammulated owl occupancy following the removal of barred owls was likely not a result of a reduction in competition. Rather, the ecological release of flammulated owls presumably occurred as a result of a reduction in either direct (consumptive) or indirect (nonconsumptive) effects of barred owls as a predator. Although no cases of barred owl predation on flammulated owls have been documented, barred owls are known to prey on and reduce the occurrence of similar‐sized forest owls, such as western screech and northern saw‐whet owls (Acker, 2012; Kryshak et al., 2022; Rugg et al., 2023). While nonconsumptive effects are difficult to demonstrate, flammulated owls exhibited increased vocalization rates in areas where barred owls were acoustically detected (most barred owl acoustic detections occurred in 2018 prior to removals), potentially signaling increased call rates for territorial defense triggered by the presence of a novel competitor, as similarly documented in spotted owls exposed to low barred owl densities (Wood, Klinck, et al., 2020). Such responses may reflect short‐term behavioral plasticity under barred owl pressure. Regardless of whether effects were consumptive or nonconsumptive, neither the early status of the barred owl invasion nor niche segregation between the two species—likely shaped in part by early barred owl presence—was sufficient to prevent negative impacts on flammulated owl occupancy. Rather, we suspect that the combined effects of small body size and nocturnal activity patterns of flammulated owls rendered them vulnerable to predation by barred owls.

**FIGURE 7:**
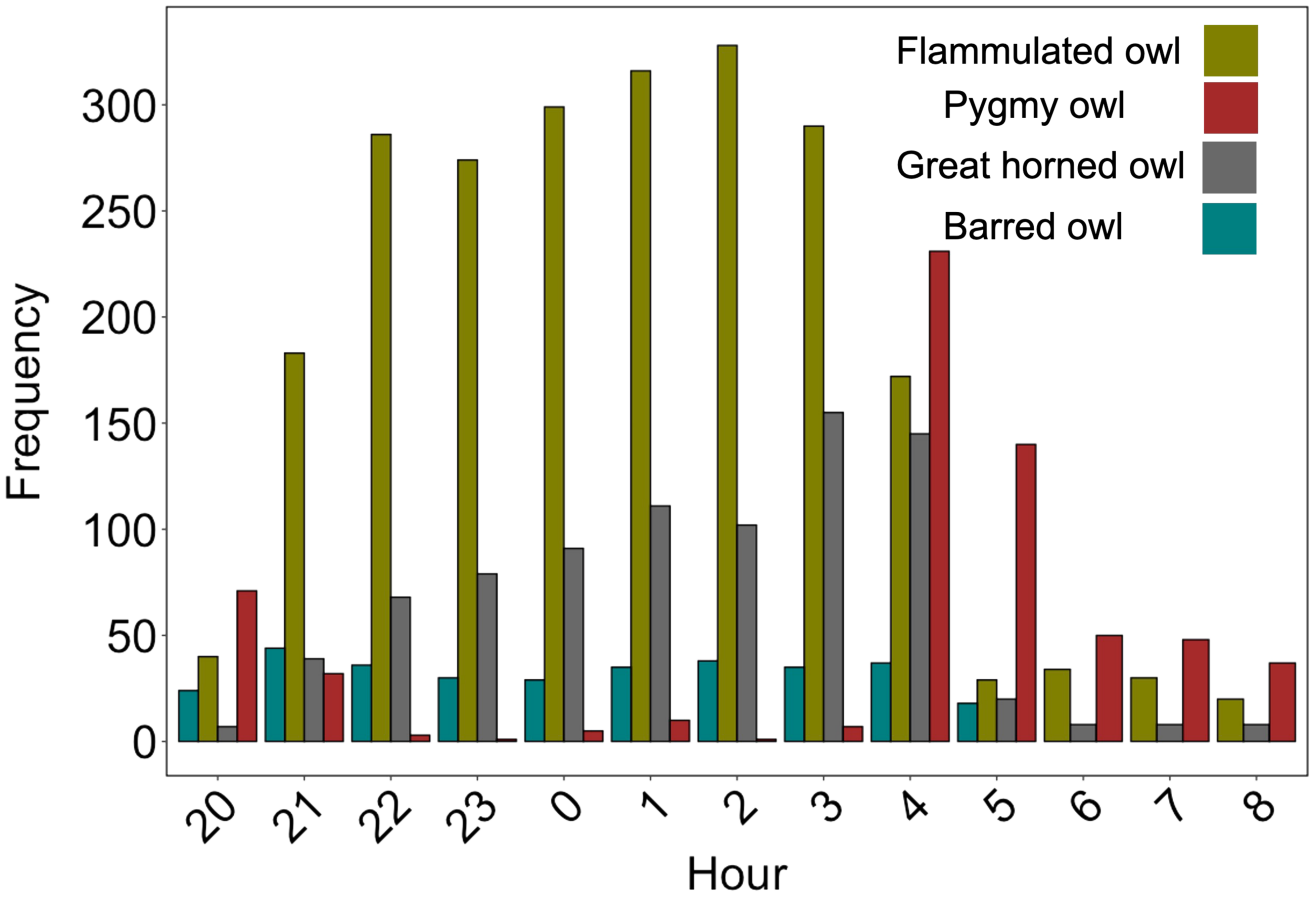
Hourly observation frequency of each native species in the northern Sierra Nevada, CA, compared to the hourly observation frequency of barred owls across the entire Sierra Nevada, CA. Barred owl detections were obtained through a passive acoustic monitoring program spanning the entire Sierra Nevada, CA (Winiarski et al., 2024).

By contrast, neither pygmy nor great horned owls increased in occupancy following experimental barred owl removals, indicating that barred owls at low densities had little effect on these two species. Both pygmy owls and great horned owls exhibited some spatial segregation with barred owls, but less than that which was observed for flammulated owls (Figure 5). Thus, spatial segregation was probably not the primary ecological mechanism preventing negative interactions between the species. Pygmy owls exhibit crepuscular and diurnal hunting behavior (Deshler et al., 2019; Sater et al., 2006), which presumably reduces their exposure to nocturnal predation by barred owls (Figure 7). As invasions progress and barred owl densities increase, co‐occurrence between the encroaching species and pygmy owls presumably increases. However, pygmy owl occupancy did not appear to increase following lethal removals in the Pacific Northwest where barred owl densities are higher (Wiens et al., Forthcoming). This suggests that the pygmy owl's diel activity reduces the barred owl's predatory threat. Consistent with our predictions, we found no evidence that barred owl removals impacted great horned owls. Great horned owls are larger than barred owls and have been observed hunting barred owls. Indeed, barred owls in their native range exhibit spatial avoidance of this larger and potentially more dominant raptor (Laidig & Dobkin, 1995).

Collectively, our results suggest that life history traits play a strong role in mediating the impacts of invasive barred owls on other forest owls. In particular, the small body size and nocturnal activity patterns of flammulated owls appear to increase their vulnerability to barred owls. In other regions, the presence of barred owls reduces the occupancy of other small‐bodied nocturnal species, such as northern saw‐whet owls and western screech owls (Acker, 2012; Rugg et al., 2023; Wiens et al., Forthcoming) but does not adversely impact pygmy owls or great horned owls (Wiens et al., Forthcoming). In the absence of barred owl removals, the structure and composition of owl communities in western forests may shift towards larger bodied species with more diurnal constituents in response to increasing barred owl density.

### Combined effects of severe wildfire and barred owl invasion

Contrary to our “interactive” hypothesis (Figure 2c), severe wildfires did not inhibit forest owl recovery after barred owl removals. Specifically, pygmy owls and great horned owls showed no significant change in occupancy and demonstrated resilience to short‐term fire effects, while flammulated owls were relatively unlikely to occur in severely burned areas, regardless of whether barred owls have been removed. However, low barred owl densities may have obscured interactions between stressors, which could emerge in areas with higher barred owl densities and extensive removal efforts. Additionally, there appears to be an interaction between barred owls and severe wildfires, as barred owls tend to avoid areas extensively burned by severe wildfires (Duchac et al., 2021; Watson et al., 2024), while some native owls appear more resilient. Where barred owls occur locally in higher densities, severe fire may indirectly benefit some native owls by reducing barred owl habitat.

### Management implications

Understanding forest owl responses to severe wildfires is important for conserving these species and managing their habitats as wildfire regimes change (Jones et al., 2016; Stevens et al., 2017). Flammulated owls face short‐term threats from increasingly large and severe fires. One such disturbance was the Dixie Fire, which burned 69% of the Lassen National Forest, reduced the availability of unburned forests at a regional scale, and created large areas of open, severely burned habitat. If managers seek to conserve and promote flammulated owls in this landscape, then management strategies should aim to conserve large trees and some live foliage, reduce the spread of high‐severity wildfire, and promote low–moderate severity fire. This approach could involve judicious use of prescribed burns and mechanical fuel treatments to retain key habitat elements. Fuels treatments may incur some cost to flammulated owls, as well as other species like spotted owls and American goshawks (*Astur atricapillus*) that rely on late seral forest characteristics (Blakey et al., 2020; Jones et al., 2020; North et al., 2017). However, the benefits of reducing or eliminating the negative impact of high‐severity fire on these species may outweigh potential costs (Blakey et al., 2020; Jones et al., 2022; Yanco & Linkhart, 2018). Moreover, in recently burned areas, preserving key postfire structures such as snags and other habitat elements utilized by woodpeckers could enhance nesting opportunities for secondary‐cavity nesters like smaller forest owls.

Given the adverse effects of high barred owl densities on some owl species reported in the literature and the increase in flammulated owl occupancy following barred owl removals at low densities, our results suggest that prompt lethal removals of the invasive owl could enable native forest owls to coexist with low barred owl densities. Early detection allows managers to act under the “Precautionary Principle,” which advocates proactive measures when activities pose potential harm to human health or the environment, even without a fully established cause‐and‐effect relationship (Kriebel et al., 2001). The conservation community invoked this principle to justify prompt barred owl lethal removals in the Sierra Nevada (Wood, Gutiérrez, et al., 2020), in light of their likely direct and indirect effects on many species (Holm et al., 2016; Kryshak et al., 2022; Wiens et al., 2021). We reiterate the application of the Precautionary Principle in guiding conservation responses to barred owls at or beyond the leading edges of their invasion, including the Sierra Nevada and the Rocky Mountains. Lethal removals are most effective when barred owl populations are sparse and localized (Diller et al., 2014; Hofstadter et al., 2022), as observed in our study area. However, reducing barred owl densities to promote coexistence with other owl species is costly, labor‐intensive, and less effective once barred owls have reached high densities (Wiens et al., 2020). Allowing barred owls to reach high densities will likely expand their ecological niche (Watson et al., 2024; Wood et al., 2021), increasing habitat overlap with native owls. Proactive removals add value amid changing wildfire regimes, as some owl species stressed by past fires face greater risks from future fires. Our study emphasizes proactive management informed by experimentation and regional, passive acoustic monitoring, offering insights for forest owl conservation and other ecosystems under invasive species and changing disturbance pressures.

## AUTHOR CONTRIBUTIONS

**Joshua M. Barry**: Conceptualization; methodology; validation; formal analysis; writing—original draft; visualization. **Connor M. Wood:** Methodology; resources; writing—review and editing. **Gavin M. Jones:** Methodology; writing—review and editing. **Kate A. McGinn:** Validation; methodology; data curation; writing—review and editing. **Kevin G. Kelly:** Data curation; writing—review and editing. **H. Anu Kramer:** Data curation; writing—review and editing. **Daniel F. Hofstadter:** Investigation; writing—review and editing. **Stefan Kahl:** Methodology; writing—review and editing. **Holger Klinck:** Methodology; writing—review and editing. **Nicholas F. Kryshak:** Investigation; writing—review and editing. **Brian P. Dotters:** Investigation; writing—review and editing. **Kevin N. Roberts:** Investigation; writing—review and editing. **John J. Keane:** Investigation; writing—review and editing. **Elizabeth Ng:** Validation; writing—review and editing. **M. Zachariah Peery:** conceptualization; methodology; resources; writing—original draft; supervision; project administration; funding acquisition.

## CONFLICT OF INTEREST STATEMENT

The authors declare no conflicts of interest.

## Supporting information


Appendix S1.

## Data Availability

Geographic locations of autonomous recording units from which detections were obtained are sensitive; these data are owned by the Department of Forest and Wildlife Ecology at the University of Wisconsin‐Madison and are available to qualified researchers by contacting the principal investigator of the Sierra Nevada Acoustic Monitoring Program (M. Zachariah Peery; email: mpeery@wisc.edu) and requesting access to the 2018 to 2023 field season metadata. Data and code (Barry et al., 2025), including anonymized encounter histories used in occupancy analyses, are publicly available in Dryad at https://doi.org/10.5061/dryad.c866t1gkt.
