# Supplementary material for: Divergent responses of native predators to severe wildfire and biological invasion are mediated by life history
Source: Ecol Appl. 2025 Nov 10;35(7):e70135. doi: 10.1002/eap.70135 (PMC12602772; doi:10.1002/eap.70135)

Appendix S1

Authors Joshua M Barry, Connor M Wood, Gavin M Jones, Kate A McGinn, Kevin G Kelly, H Anu Kramer, Daniel F Hofstadter, Stefan Kahl, Holger Klinck, Nicholas F Kryshak, Brian P Dotters, Kevin N Roberts, John J Keane, Elizabeth Ng, and M Zachariah Peery

Title Divergent responses of native predators to severe wildfire and biological invasion are mediated by life history

Journal Ecological Applications

Table S1. The set count of observations per hour-long file that have been selected, the set prediction score thresholds, and the resultant number of sites featuring detections meeting these defined criteria. We also report naïve occupancy (not corrected for detection).

Species	Year	1% Threshold		Manual Threshold				
		Count	Prediction score	Count	Prediction score	FP Rate[a]	TP Sites[b]	Naïve occupancy
Flammulated owl	2023	155	0.90	10	0.90	42%	98	0.24
	2022	155	0.90	10	0.90	42%	80	0.17
	2021	155	0.90	10	0.90	42%	93	0.18
	2018	45	0.50	8	0.70	42%	154	0.30
Pygmy owl	2023	150	0.99	5	0.90	15%	39	0.10
	2022	150	0.99	5	0.90	15%	65	0.14
	2021	150	0.99	5	0.90	15%	112	0.22
	2018	22	0.50	5	0.70	13%	81	0.16
Great horned owl	2023	34	0.90	N/A	N/A	N/A	34	0.16
	2022	34	0.90	N/A	N/A	N/A	41	0.16
	2021	34	0.90	N/A	N/A	N/A	57	0.22
	2018	10	0.90	N/A	N/A	N/A	92	0.35
Western screech-owl	2023	16	0.99	N/A	N/A	N/A	29	0.06
	2022	16	0.99	50	0.93	49%	32	0.06
	2021	16	0.99	50	0.93	49%	40	0.08
	2018	51	0.95	20	0.10	86%	27	0.05
Saw-whet owl	2023	140	0.90	N/A	N/A	N/A	2	<0.01
	2022	140	0.90	10	0.90	25%	20	0.04
	2021	140	0.90	10	0.90	25%	16	0.03
	2018	96	0.50	15	0.10	7%	46	0.09

[a] False positive rate of hourly detection.
[b] The site level for flammulated owls, northern pygmy owls, western screech-owls, and saw-whet owls was represented as the individual autonomous recording units, whereas the site level for great horned owls was represented as the 400-ha hexagonal cell.

Table S2. Posterior estimates and confidence intervals (85%) of model coefficients for wildfire occupancy models accounting for relative effects. Target species included flammulated owls, northern pygmy owls, and great horned owls. The fire model covariates included sites burned at high severity (=burn), and the proportional amount of severe fire one- (=burn1), two- (=burn2), three-years (=burn3), and all years (=allpostburn) post-fire.

Predictor	Species	Model	Covariate	Estimate	85% CLs Lower	85% CLs Upper	Non-zero overlap	% Posteriors < 0
Severe fire	FLM	Ψ ($burn_i + burn1_i + burn2_i + burn3_i$)	$burn_i$	0.99	0.59	1.41	Yes	0.0
	FLM	Ψ ($burn_i + burn1_i + burn2_i + burn3_i$)	$burn1_i$	-0.98	-1.91	-0.08	Yes	93.9
	FLM	Ψ ($burn_i + burn1_i + burn2_i + burn3_i$)	$burn2_i$	-2.08	-3.14	-1.03	Yes	99.8
	FLM	Ψ ($burn_i + burn1_i + burn2_i + burn3_i$)	$burn3_i$	-1.28	-2.61	-0.01	Yes	92.6
	FLM	Ψ ($burn_i + allpostburn_i$)	$allpostburn_i$	-1.71	-2.56	-0.92	Yes	99.9
	NPO	Ψ ($burn_i + burn1_i + burn2_i + burn3_i$)	$burn_i$	-0.26	-0.55	0.03	No	90.6
	NPO	Ψ ($burn_i + burn1_i + burn2_i + burn3_i$)	$burn1_i$	0.24	-0.49	0.97	No	31.6
	NPO	Ψ ($burn_i + burn1_i + burn2_i + burn3_i$)	$burn2_i$	-0.25	-1.17	0.64	No	65.6
	NPO	Ψ ($burn_i + burn1_i + burn2_i + burn3_i$)	$burn3_i$	-0.20	-1.41	0.96	No	59.6
	NPO	Ψ ($burn_i + allpostburn_i$)	$allpostburn_i$	0.03	-0.59	0.65	No	47.4
	GHO	Ψ ($burn_i + burn1_i + burn2_i + burn3_i$)	$burn_i$	0.56	0.06	1.05	Yes	5.3
	GHO	Ψ ($burn_i + burn1_i + burn2_i + burn3_i$)	$burn1_i$	-0.02	-1.20	1.15	No	50.4
	GHO	Ψ ($burn_i + burn1_i + burn2_i + burn3_i$)	$burn2_i$	-0.66	-2.00	0.63	No	76.9
	GHO	Ψ ($burn_i + burn1_i + burn2_i + burn3_i$)	$burn3_i$	-0.58	-2.33	1.04	No	68.6
	GHO	Ψ ($burn_i + allpostburn_i$)	$allpostburn_i$	-0.50	-1.51	0.51	No	76.5

Abbreviations: FLM, flammulated owl; NPO, northern pygmy owl; GHO, great horned owl.

Table S3. Posterior estimates and confidence intervals (85%) of model coefficients for barred owl removal occupancy models accounting for relative effects. Target species included flammulated owls, northern pygmy owls, and great horned owls. The barred owl model covariates included barred owl lethal removals (=site-type), one-year post-removal at removal sites (=lethal1), two-years post-removal at removal sites (=lethal2), three or more years post-removal at removal sites (=lethal3+), and all post-lethal removal sites regardless of time since removal (=allpostlethal).

Predictor	Species	Model	Covariate	Estimate	85% CLs Lower	85% CLs Upper	Non-zero overlap	% Posteriors < 0
Barred owl	FLM	Ψ (*site-type$_i$* + *lethal1$_i$* + *lethal2$_i$* + *lethal3+$_i$*)	*site-type$_i$*	-1.14	-1.96	-0.34	Yes	98.0
	FLM	Ψ (*site-type$_i$* + *lethal1$_i$* + *lethal2$_i$* + *lethal3+$_i$*)	*lethal1$_i$*	0.90	-0.61	2.44	No	19.6
	FLM	Ψ (*site-type$_i$* + *lethal1$_i$* + *lethal2$_i$* + *lethal3+$_i$*)	*lethal2$_i$*	0.43	-0.57	1.40	No	26.7
	FLM	Ψ (*site-type$_i$* + *lethal1$_i$* + *lethal2$_i$* + *lethal3+$_i$*)	*lethal3+$_i$*	0.76	-0.13	1.66	No	10.9
	FLM	Ψ (*site-type$_i$* + *allpostlethal$_i$*)	*site-type$_i$*	-1.25	-2.07	-0.45	Yes	97.9
	FLM	Ψ (*site-type$_i$* + *allpostlethal$_i$*)	*allpostlethal$_i$*	0.86	0.04	1.70	Yes	6.4
	NPO	Ψ (*site-type$_i$* + *lethal1$_i$* + *lethal2$_i$* + *lethal3+$_i$*)	*site-type$_i$*	-0.32	-0.90	0.25	No	78.6
	NPO	Ψ (*site-type$_i$* + *lethal1$_i$* + *lethal2$_i$* + *lethal3+$_i$*)	*lethal1$_i$*	-1.52	-3.68	0.28	No	87.4
	NPO	Ψ (*site-type$_i$* + *lethal1$_i$* + *lethal2$_i$* + *lethal3+$_i$*)	*lethal2$_i$*	0.56	-0.17	1.28	No	13.7
	NPO	Ψ (*site-type$_i$* + *lethal1$_i$* + *lethal2$_i$* + *lethal3+$_i$*)	*lethal3$_i$*	0.20	-0.53	0.91	No	35.3
	NPO	Ψ (*site-type$_i$* + *allpostlethal$_i$*)	*site-type$_i$*	-0.27	-0.87	0.33	No	74.9
	NPO	Ψ (*site-type$_i$* + *allpostlethal$_i$*)	*allpostlethal$_i$*	0.26	-0.39	0.91	No	28.4
	GHO	Ψ (*site-type$_i$* + *lethal1$_i$* + *lethal2$_i$* + *lethal3+$_i$*)	*site-type$_i$*	-0.45	-1.21	0.32	No	80.2
	GHO	Ψ (*site-type$_i$* + *lethal1$_i$* + *lethal2$_i$* + *lethal3+$_i$*)	*lethal1$_i$*	-0.51	-2.08	0.99	No	67.2
	GHO	Ψ (*site-type$_i$* + *lethal1$_i$* + *lethal2$_i$* + *lethal3+$_i$*)	*lethal2$_i$*	-0.77	-1.81	0.24	No	85.8
	GHO	Ψ (*site-type$_i$* + *lethal1$_i$* + *lethal2$_i$* + *lethal3+$_i$*)	*lethal3$_i$*	-0.29	-1.23	0.61	No	67.6
	GHO	Ψ (*site-type$_i$* + *allpostlethal$_i$*)	*site-type$_i$*	-0.33	-1.13	0.47	No	71.9
	GHO	Ψ (*site-type$_i$* + *allpostlethal$_i$*)	*allpostlethal$_i$*	-0.62	-1.50	0.23	No	85.4

Abbreviations: FLM, flammulated owl; NPO, northern pygmy owl; GHO, great horned owl.

Table S4. Niche breadth and niche overlap estimates for native forest owls and invasive barred owls in northern California, USA. Target species include flammulated owls, northern pygmy owls, great horned owls, and barred owls.

		Two-dimensional ellipse			
Comparision	Species	Ruggedness + Elevation	Intermediate-to-late seral forests + Elevation	Intermediate-to-late seral forests + Ruggedness	Average
Niche overlap with barred owls	FLM	0.75	0.55	0.41	0.57
	GHO	0.93	0.58	0.56	0.69
	NPO	0.73	0.57	0.47	0.59
Niche Breadth	FLM	0.51	0.42	0.54	0.49
	GHO	0.39	0.38	0.39	0.39
	NPO	0.54	0.41	0.49	0.48
	BDOW	0.43	0.23	0.24	0.30

Abbreviations: FLM, flammulated owl; NPO, northern pygmy owl; GHO, great horned owl; BDOW, barred owl

Figure S1. Distribution of burned sites burned at different proportional amounts of severe wildfire in the northern Sierra Nevada, California.

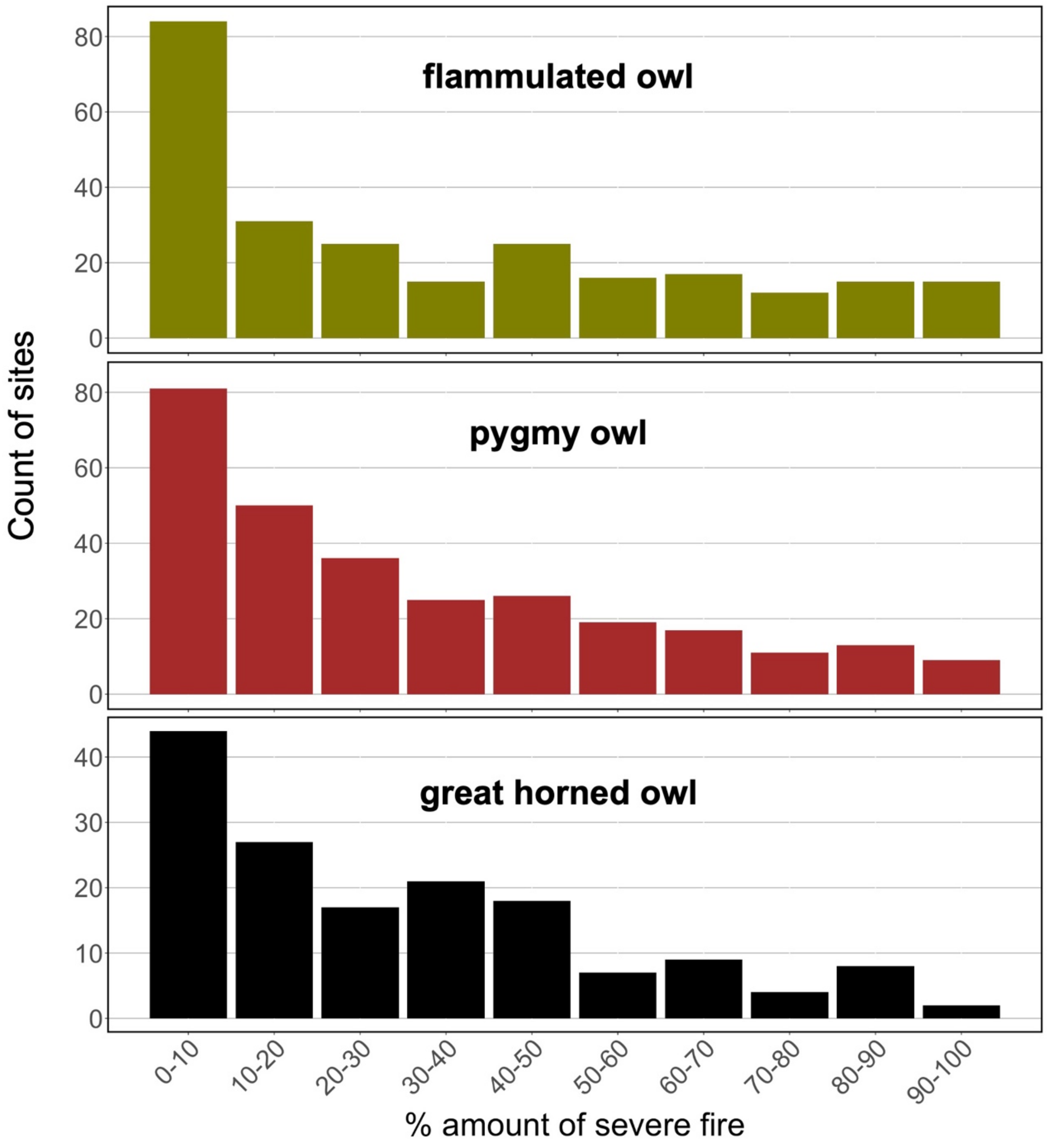

Figure S2. Posterior distributions on the logit scale for barred owl model coefficients of the target species. The target species included (a) flammulated owls, (b) northern pygmy owls, and (c) great horned owls. For flammulated owls, the percent overlap between site-type (removal sites) and 1-year, 2-years, 3+ years, and all years post removal was 11%, 12%, 6%, and 4%, respectively. For northern pygmy owls, the percent overlap between site-type (removal sites) and 1-year, 2-years, 3+ years, and all years post removal was 25%, 20%, 40%, and 37%, respectively. For great horned owls, the percent overlap between site-type (removal sites) and 1-year, 2-years, 3+ years, and all years post removal was 51%, 63%, 75%, and 67%, respectively.

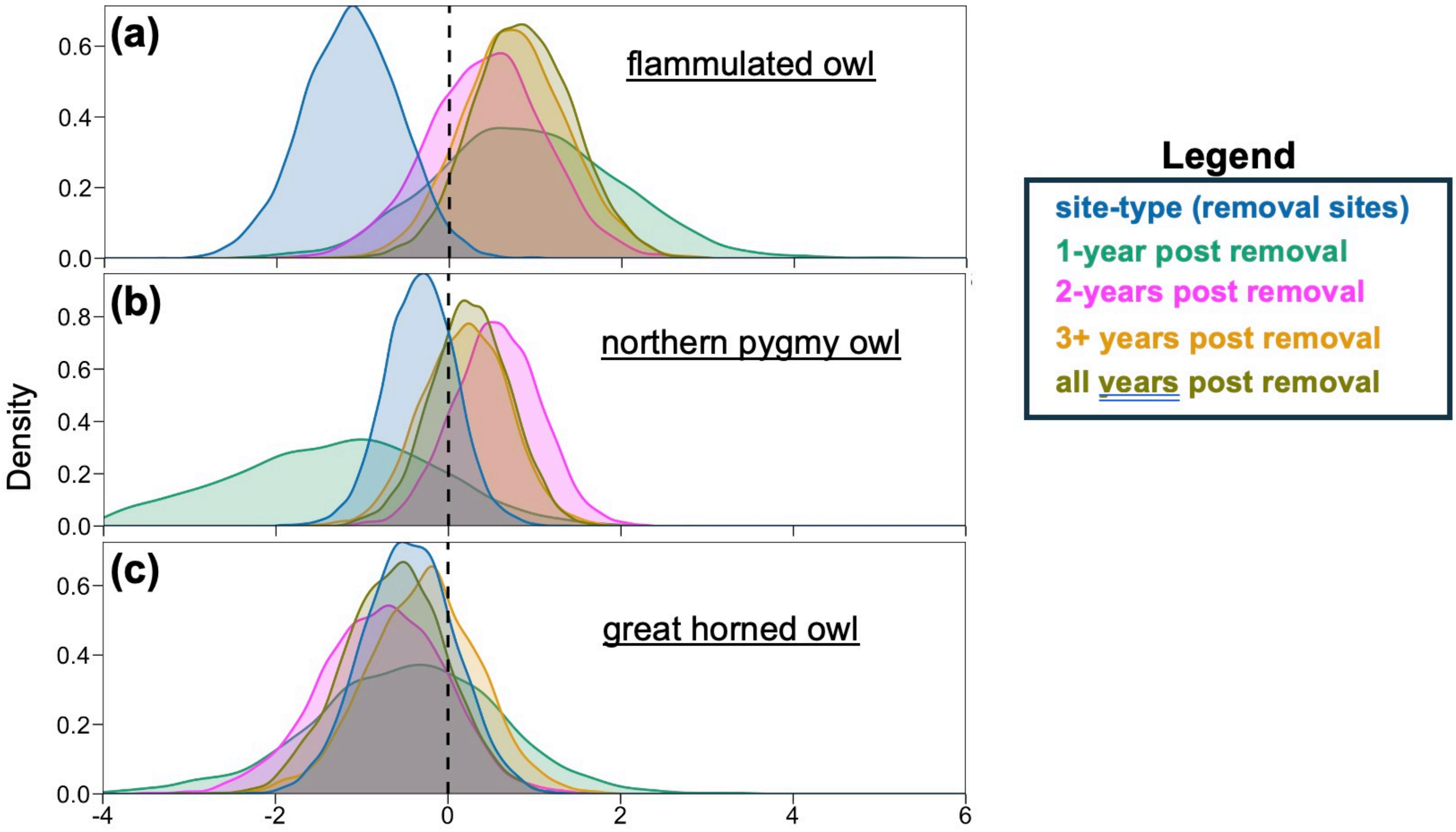

Figure S3. MacKenzie-Bailey chi-square posterior predictive check for all flammulated owl models, including the distribution of simulated and observed points. Models include the (a) time-dependent fire model, (b) the time-constant fire model, (c) the time-dependent barred owl model, and (d) the time-constant barred owl model.

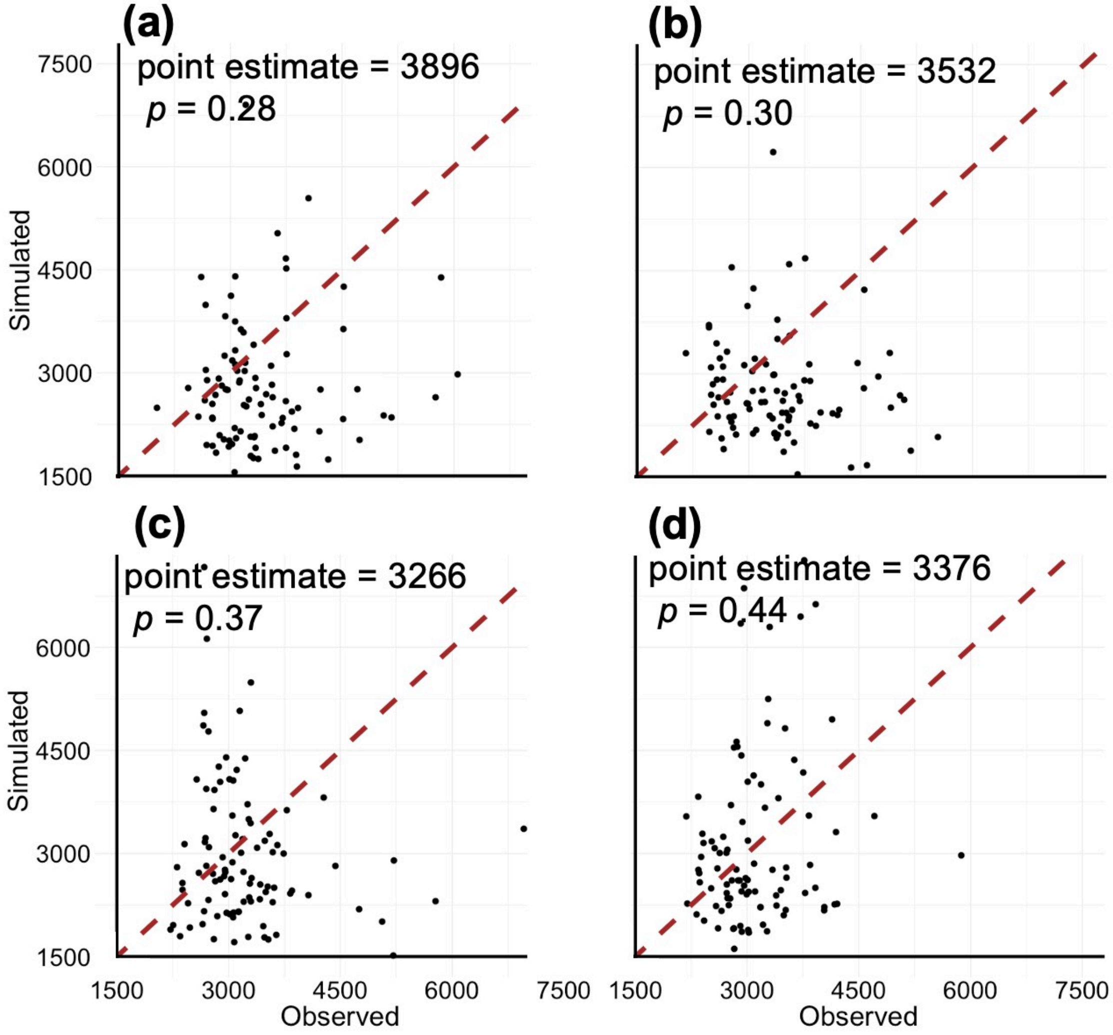

Figure S4. Traceplots of beta estimate convergence via Markov Chain Monte Carlo iterations in the flammulated owl (a-e) time-dependent wildfire model and the (f) time-constant wildfire model.

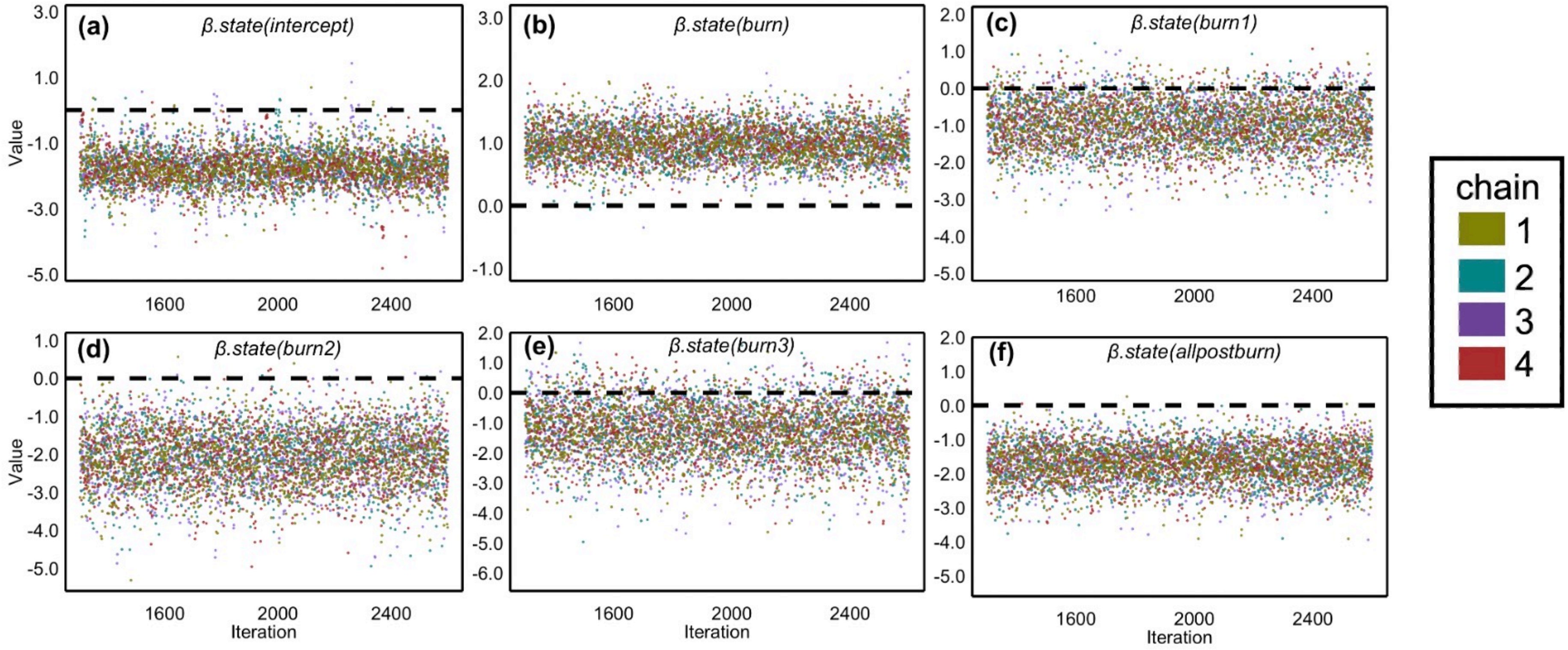

Figure S5. Forest owl detections in the northern Sierra Nevada, California, using regional-scale, passive acoustic surveys from 2018 to 2023, both before (light grey circles) and after (dark grey circles) the lethal removal of barred owls and two severe fire events. Target species include flammulated (panels a - d), northern pygmy (e – h), and great horned owls (i – l). Detections are indicated at the hexagonal cell level.

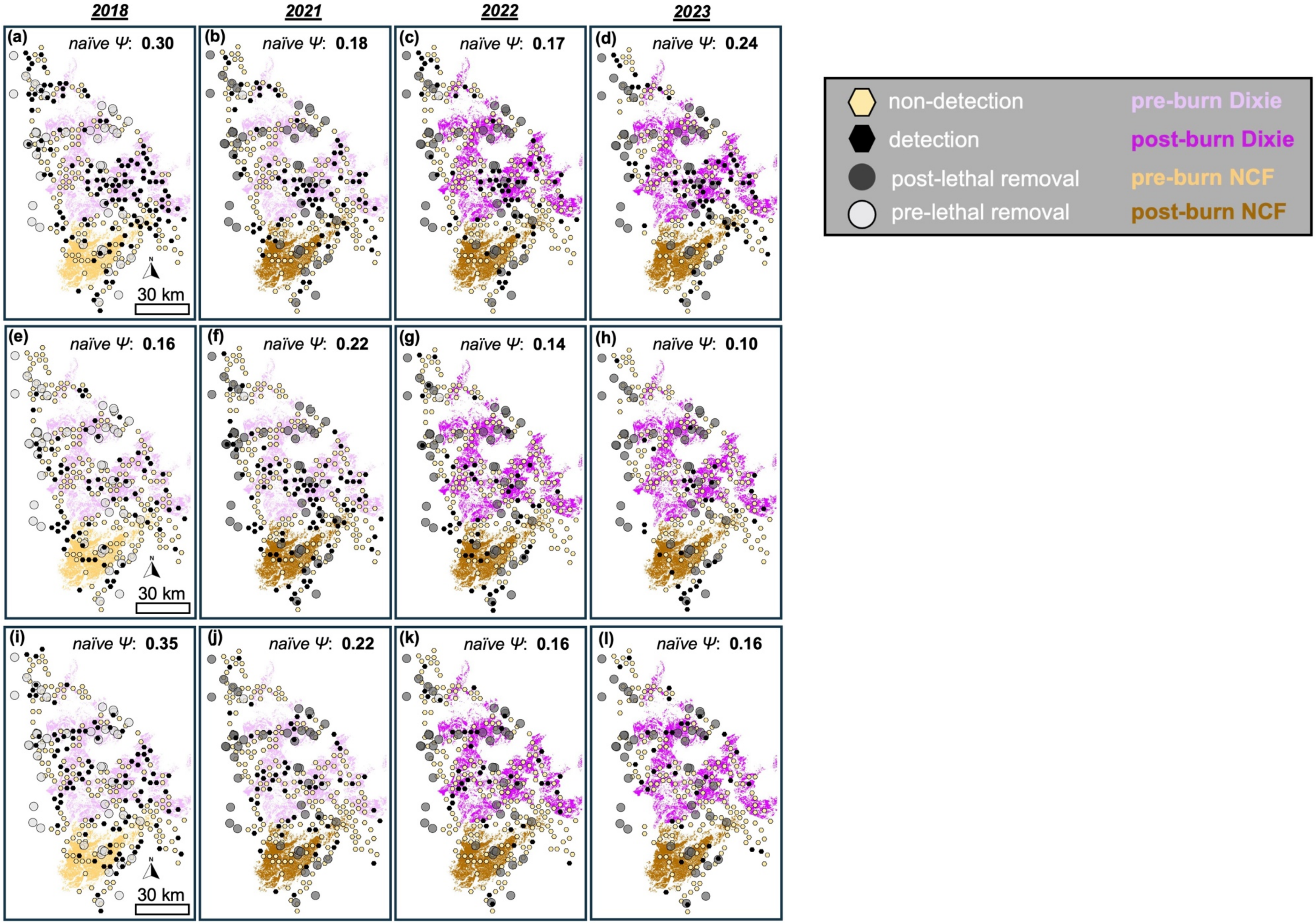

Figure S6. Estimated site occupancy (with 85% credible intervals) for (a-c) flammulated owls, (d-f) northern pygmy owls, and (g-i) great horned owls one, two, and three years after severe wildfires.

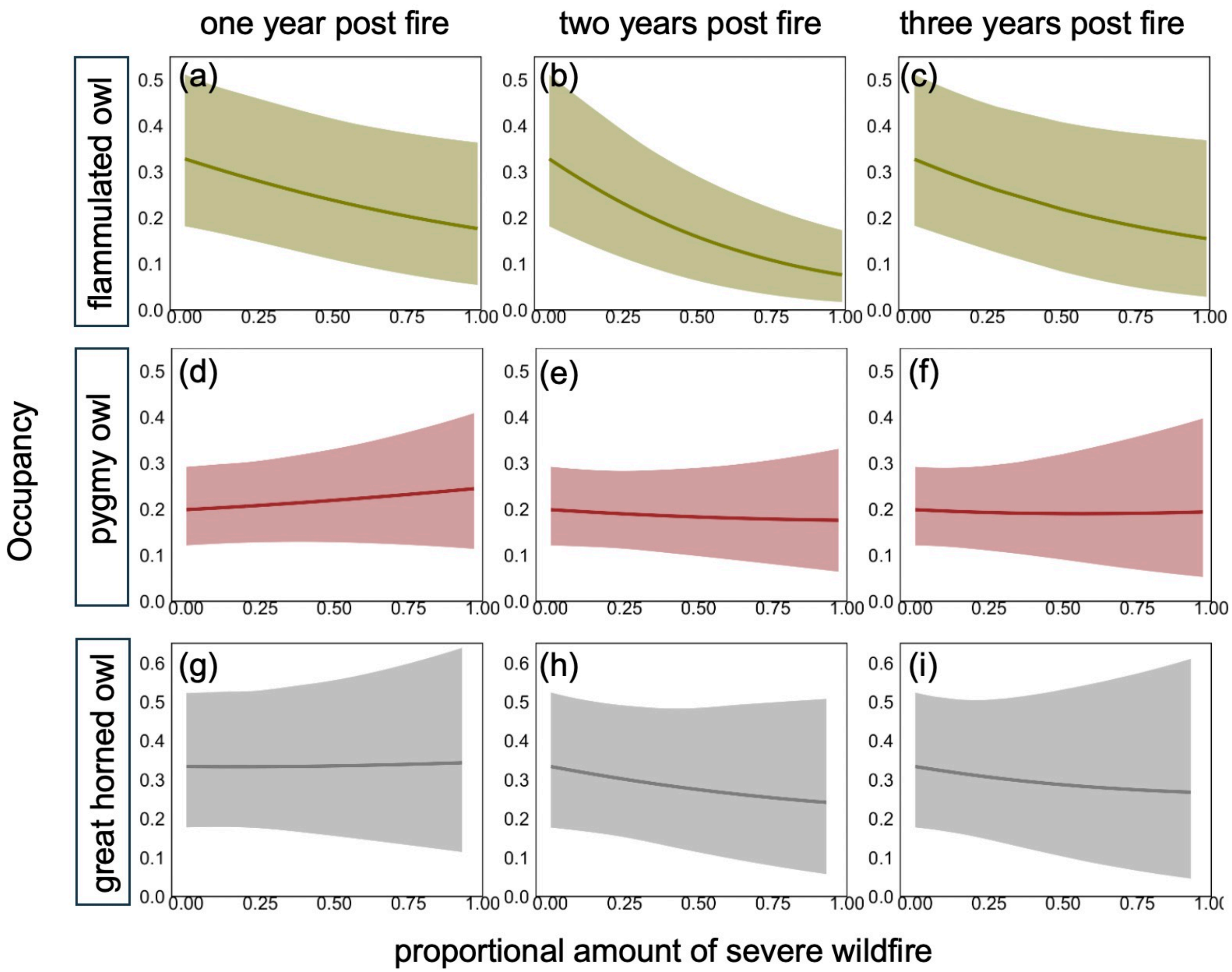

Figure S7. Mean estimated site occupancy (with 85% credible intervals) for (a) flammulated owls, (b) northern pygmy owls, and (c) great horned owls at control sites (=dashed line), removal sites pre-removal, and removal sites one, two, or three or more years after lethal barred owl removals.

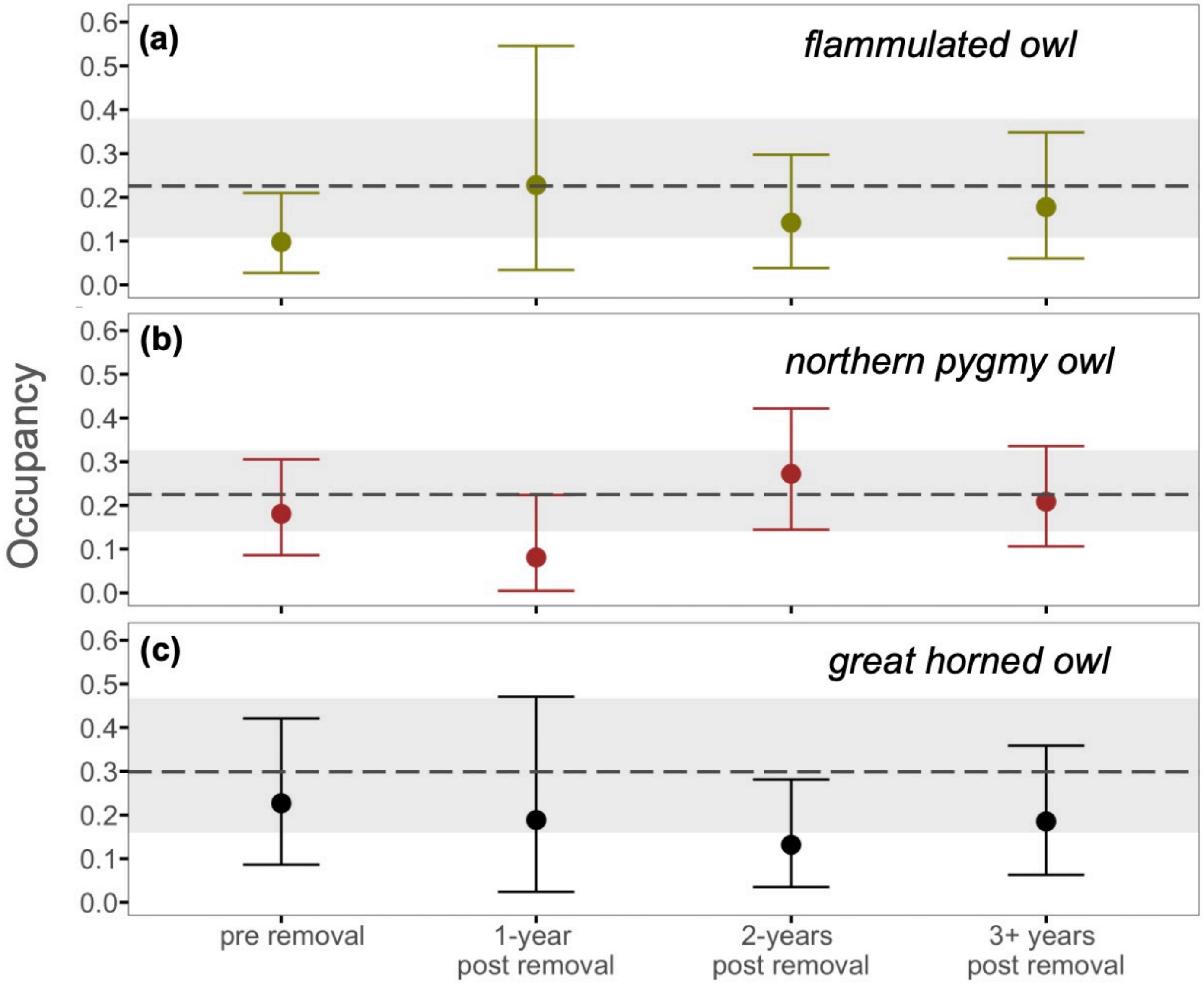

Figure S8. Distribution of target owl species in 2018 with elevation and barred owl removal and detection locations.

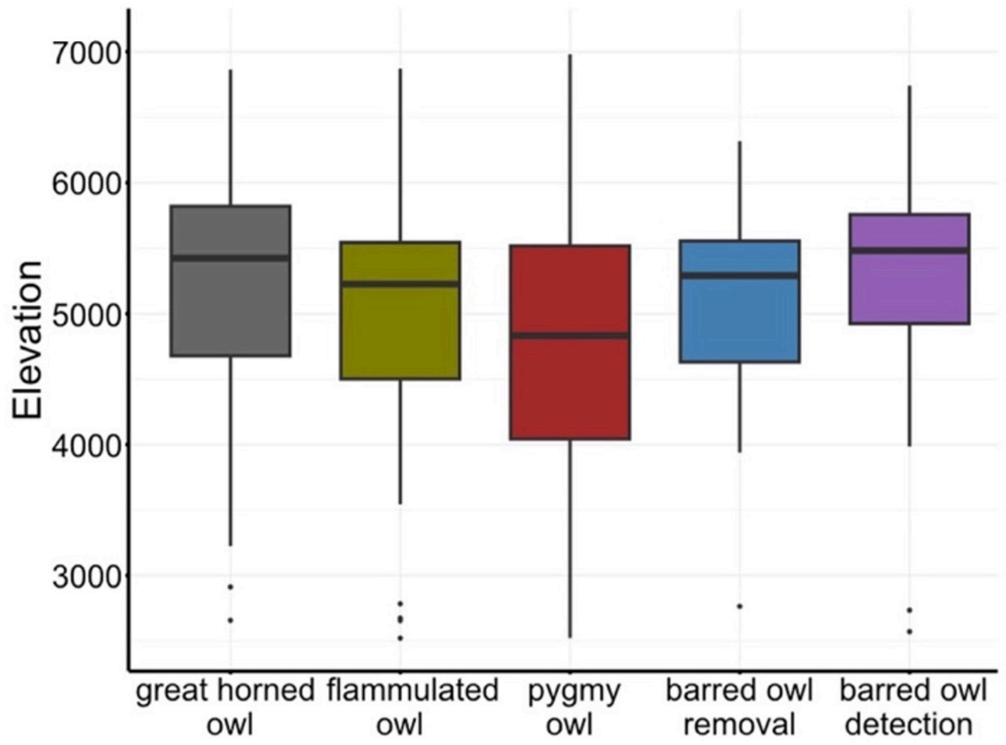

Figure S9. Relationship between the proportional amount of intermediate-to-late seral forests and probability of barred owl occurrence in the northern Sierra Nevada, California, USA. The plot shows the probabilities of occurrence for both detected (n = 67) and non-detected (n = 234) sites.

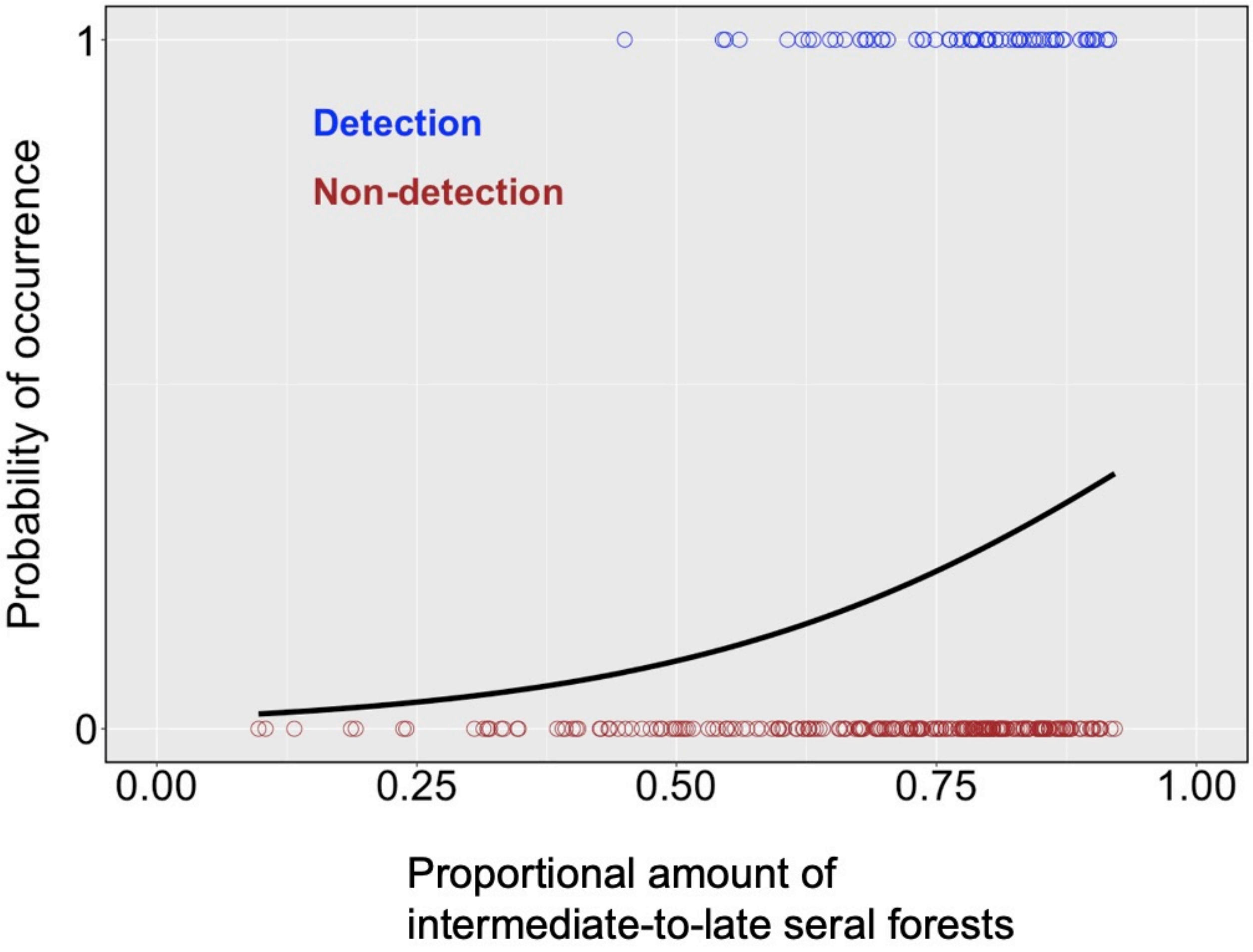

Supplement: Supplementary file 1 — Appendix S1. [file EAP-35-e70135-s001.pdf]
